# Base-Pair Resolution DNA Methylation Sequencing Reveals Profoundly Divergent Epigenetic Landscapes in Acute Myeloid Leukemia

**DOI:** 10.1371/journal.pgen.1002781

**Published:** 2012-06-21

**Authors:** Altuna Akalin, Francine E. Garrett-Bakelman, Matthias Kormaksson, Jennifer Busuttil, Lu Zhang, Irina Khrebtukova, Thomas A. Milne, Yongsheng Huang, Debabrata Biswas, Jay L. Hess, C. David Allis, Robert G. Roeder, Peter J. M. Valk, Bob Löwenberg, Ruud Delwel, Hugo F. Fernandez, Elisabeth Paietta, Martin S. Tallman, Gary P. Schroth, Christopher E. Mason, Ari Melnick, Maria E. Figueroa

**Affiliations:** 1 Department of Physiology and Biophysics and the HRH Prince Alwaleed Bin Talal Bin Abdulaziz Alsaud Institute for Computational Biomedicine, Weill Cornell Medical College, New York, New York, United States of America; 2 Department of Medicine, Division of Hematology/Oncology, Weill Cornell Medical College, New York, New York, United States of America; 3 Department of Public Health, Weill Cornell Medical College, New York, New York, United States of America; 4 Illumina, Hayward, California, United States of America; 5 MRC Molecular Haematology Unit, Weatherall Institute of Molecular Medicine, University of Oxford, Oxford, United Kingdom; 6 Department of Pathology, University of Michigan, Ann Arbor, Michigan, United States of America; 7 Laboratory of Chromatin Biology, The Rockefeller University, New York, New York, United States of America; 8 Laboratory of Molecular Biology and Biochemistry, The Rockefeller University, New York, New York, United States of America; 9 Department of Hematology, Erasmus University Medical Center, Rotterdam, The Netherlands; 10 Moffitt Cancer Center and Research Institute, Tampa, Florida, United States of America; 11 Cancer Center, Montefiore Medical Center–North Division, Bronx, New York, United States of America; 12 Leukemia Service, Memorial Sloan-Kettering Cancer Center, New York, New York, United States of America; 13 Department of Pharmacology, Weill Cornell Medical College, New York, New York, United States of America; Cincinnati Children's Hospital Medical Center, United States of America

## Abstract

We have developed an enhanced form of reduced representation bisulfite sequencing with extended genomic coverage, which resulted in greater capture of DNA methylation information of regions lying outside of traditional CpG islands. Applying this method to primary human bone marrow specimens from patients with Acute Myelogeneous Leukemia (AML), we demonstrated that genetically distinct AML subtypes display diametrically opposed DNA methylation patterns. As compared to normal controls, we observed widespread hypermethylation in IDH mutant AMLs, preferentially targeting promoter regions and CpG islands neighboring the transcription start sites of genes. In contrast, AMLs harboring translocations affecting the *MLL* gene displayed extensive loss of methylation of an almost mutually exclusive set of CpGs, which instead affected introns and distal intergenic CpG islands and shores. When analyzed in conjunction with gene expression profiles, it became apparent that these specific patterns of DNA methylation result in differing roles in gene expression regulation. However, despite this subtype-specific DNA methylation patterning, a much smaller set of CpG sites are consistently affected in both AML subtypes. Most CpG sites in this common core of aberrantly methylated CpGs were hypermethylated in both AML subtypes. Therefore, aberrant DNA methylation patterns in AML do not occur in a stereotypical manner but rather are highly specific and associated with specific driving genetic lesions.

## Author Summary

Acute myeloid leukemias (AML) are a group of malignancies that originate in the bone marrow. While many different genetic lesions have been linked to the different forms of this disease, it is also clear that these genetic lesions are not always sufficient to cause AML. DNA methylation plays a role in gene expression regulation, and abnormal distribution of DNA methylation has been observed in many cancers, including AML. Here we demonstrate that changes in DNA methylation in AML are not uniform across all AML subtypes, but rather they display unique patterns, which are closely linked to the underlying genetic lesions of each of the different forms of AML. Furthermore, these unique patterns of DNA methylation have different impacts on gene expression regulation in each AML subtype.

## Introduction

Acute myeloid leukemia (AML) is considered to be a genetically heterogeneous group of diseases, featuring functionally distinct somatic mutations and chromosomal translocations [1]. Many of these mutations involve aberrant transcriptional and epigenetic regulators, such as translocations involving chromosome 11q23, which fuse the N-terminal portion of the Mixed Lineage Leukemia protein (MLL) to various fusion partners. MLL fusion proteins feature aberrant chromatin modifying functions and drive leukemogenesis through aberrant transcriptional activation of target genes such as *HOXA9* [2]–[4]. More recently, AML associated heterozygous somatic mutations of isocitrate dehydrogenase 1 or 2 (IDH1 or 2) were shown to result in a gain of function enzyme that uses alpha-ketoglutarate (αKG) as a substrate to generate the oncometabolite 2-hydroxyglutarate (2HG) [5]. Accumulation of 2HG inhibits the function of αKG-dependent enzymes including the TET family of dioxygenases [6]–[8]. TET proteins contribute to DNA demethylation by hydroxylating 5-methylcytosine (5mC) [9]. 2HG-induced suppression of TET proteins leads to accumulation of DNA methylation with effects on epigenetic gene regulation [10].

DNA methylation profiling of AMLs indicate that disruption of promoter cytosine methylation patterning is a universal feature of the disease. Promoter methylation signatures identify AML as composed of sixteen epigenetically defined subtypes [11]. One of these epigenetically defined AML subtypes feature 11q23 translocations and another features IDH1/2 somatic mutations. Indeed, abnormal promoter methylation has been noted in several other cancers. Recent more comprehensive DNA methylation sequencing studies indicate that cancers display perturbed cytosine methylation compared to normal tissues either on the basis of changes in CpG island methylation or alternatively at CpG shores, and have offered partially different visions of how DNA methylation is perturbed in tumor cells, in part influenced by technical differences in methods used to capture this information [12]–[14]. However, direct and quantitative genome scale studies of cytosine methylation perturbation in the context of tumors with specific genetic backgrounds have not been published for any cancer. Hence it is not known whether epigenetic patterning in cancer has a stereotypical pattern with a subtext of certain promoter specific aberrancies, or whether epigenetic patterning is mechanism and tumor subtype specific. One practical way to approach this question is through reduced representation bisulfite sequencing (RRBS), an efficient method for quantitative, base-pair resolution of cytosine methylation across the genome [15], [16]. However, this procedure has been shown to mainly represent CpG islands at the expense of other genomic regions [17], [18]. In order to address the question of whether DNA methylation patterning is stereotyped or mechanism specific in tumors, we established an enhanced RRBS procedure (ERRBS) that provides biochemical and bioinformatic methodological improvements that generate more extensive and balanced coverage of CpGs. ERRBS analysis of normal hematopoietic stem cells in comparison with MLL rearranged (MLLr) or IDH1/2 mutant (IDH-mut) AMLs reveals that DNA methylation patterning is established in a profoundly distinct and mechanism specific manner in AMLs.

## Results

### Expansion of RRBS for enhanced coverage of genomic CpG methylation

We sought to examine quantitative, base-pair resolution DNA methylation patterns in clinical specimens with limited cell numbers, with adequate coverage of CpGs both within and outside of CpG islands. To accomplish this, we developed a modified version of the RRBS protocol, which retains its quantitative base-pair resolution while improving the coverage of regions outside CpG islands. We first validated the performance of the original RRBS assay using genomic DNA extracted from the HCT116 colon cancer cell line. We observed that RRBS yielded robust and reproducible results over a wide range of starting material ranging from 5 ng to 1000 ng (Figure S1A) without any significant sequencing strand bias (Figure S1B).

We next modified RRBS into a format that would be practical to perform in limited clinical specimens. First, we eliminated an intermediate clean-up procedure between the two rounds of bisulfite treatment in order to minimize sample loss during library preparation. Instead of two rounds of bisulfite conversion as previously described [16], [19] we used just one 16-hour round using the EZ DNA Methylation Kit (Zymo Research, CA) with slight modifications to the manufacturer's suggested protocol (see methods section). This approach routinely achieves a conversion rate greater than 99.8% in both human and murine samples (Table S1). Conversion rates >99% with RRBS have been observed but not consistently achieved and rarely surpass 99.5%, even with repeated rounds of bisulfite conversion [16].

While RRBS has been shown to reliably detect gain of methylation, its ability to accurately detect genome-wide loss of methylation has not been extensively probed. Yet this is essential for clinical samples, since aberrant hypomethylation can be a dominant feature of tumor cells [7], [11], [12], [20]. Furthermore, DNA methyltransferase (DNMT) inhibitor drugs currently used in the clinic are capable of inducing extensive hypomethylation [21]. In order to examine the dynamic range of the RRBS, we compared and contrasted the methylomes of HCT116 cells and the related cell line HCT116-DKO clone 2 (DKO2) which lacks DNMT1 and DNMT3b [22]. DNA methylation in HCT116 showed the expected bimodal distribution, with the vast majority of CpG sites in the 0–10% and 90–100% methylation range (Figure S1B). In contrast, the DKO2 cell line had a unimodal peak containing >83% of the reads with levels of methylation of 0–10%. Only 5.5% of DKO reads displayed >50% methylation. (Figure S1C). Even under these extreme hypomethylated conditions the modified bisulfite treatment protocol continued to perform robustly (conversion rate = 99.9%). We further validated the accuracy of the ERRBS assay with MassArray Epityping at 45 individual CpG sites, showing an extremely high degree of correlation (r = 0.97) (Figure S1D).

**Figure 1:**
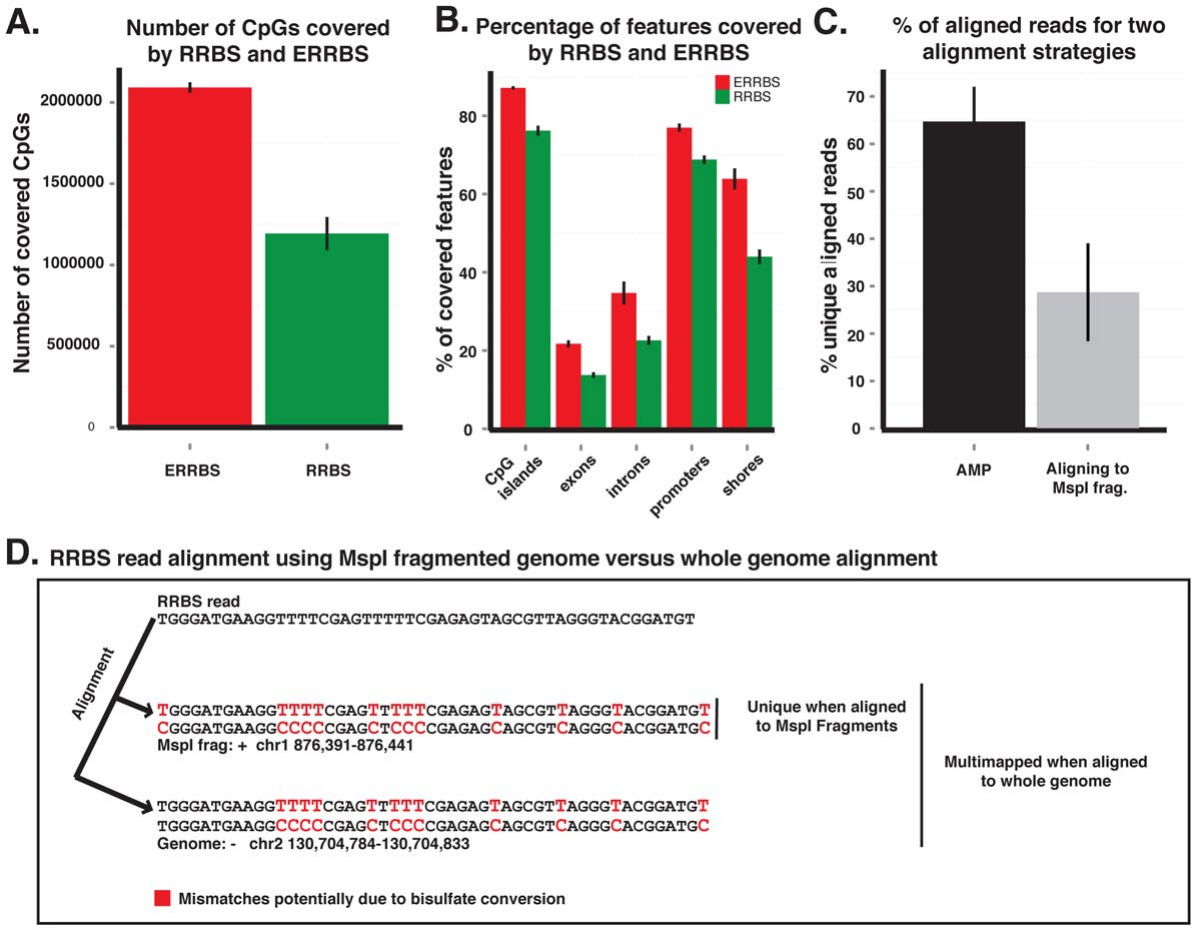
ERRBS improves genomic coverage and alignment accuracy. (A) Average CpG number coverage for ERRBS (red) and RRBS (green) methods (n = 3, samples NBM_#2, AML and MLLr_#1). (B) Average percent coverage of different genomic regions by ERRBS (red) and RRBS (green) (n = 3, samples NBM_#2, AML and MLLr_#1) (C) Average percentage of uniquely aligned reads using a whole genome reference strategy (black) or an MspI in silico digested genome reference (gray) (n = 4, samples NBM_#2, AML_Rep#2, MLLr_#1 and MLLr_#2) (D) Example of a misalignment due to the use of a reduced representation bisulfite converted reference genome. The read aligns to a unique genomic location using the MspI alignment algorithm (forward strand, chr1: 876391–876441), however the same fragment does not align uniquely when using a whole genome alignment algorithm, rather it also aligns to the reverse strand of chr 2: 130,704,784–130,704,833.

An increasing body of evidence demonstrates that biologically relevant changes in DNA methylation in cancer occur beyond CpG islands [12], [13], [23]. Because RRBS only interrogates CpGs within short MspI delimited fragments between 40 to 220 bp, it is inherently biased towards representing CpG islands, which typically contain more densely clustered MspI sites [17], [18]. In order to enhance the capture of regions beyond CpG islands, MspI fragments ranging from 70–320 bp were selected instead. This enhanced RRBS (ERRBS) method yielded a 75% increase in coverage of CpG sites with a 54% increase in coverage of CpG shores (defined as 2000 bp flanks on upstream and downstream of CpG islands). We also observed a 58% increase in the number of introns captured vs. RRBS, a 54% increase in the number of exons and an 11.9% increase in the number of promoter regions (Figure 1A and 1B).

While the original RRBS alignment strategy used an MspI fragmented genome as a reference, whole-genome alignment strategies can also be applied to these data [18]. In a direct comparison of both strategies, we observed that a whole-genome alignment approach using the Bowtie aligner via the Bismark software [24] more than doubled the number of aligned reads which resulted in an increased recovery of the number of CpGs (mean increase 200,154+/−135,012) (Figure 1C). Eliminating the use of an MspI site as the absolute alignment requirement at the beginning of reads, as well as the use of a longer (32 bp) seed length, further improved accuracy by excluding those reads that had the potential for more than one unique match or mismatch (Figure 1D). Theoretically, no reads should map to regions of the genome that are not flanked by an MspI restriction sites, yet we found that on average 29% of the aligned reads mapped to non-MspI fragments. These fragments, which would be discarded when using in silico digested genomes for alignment, were likely produced due to either restriction enzyme non-specific activity, the presence of partially degraded DNA at the onset of the protocol, or variations in the patient genome compared to the reference genome. Collectively these approaches enhanced not only genomic coverage, but also alignment efficiency and accuracy.

### Tumor subtype–specific DNA methylation patterns extend beyond promoter regions

**Figure 2:**
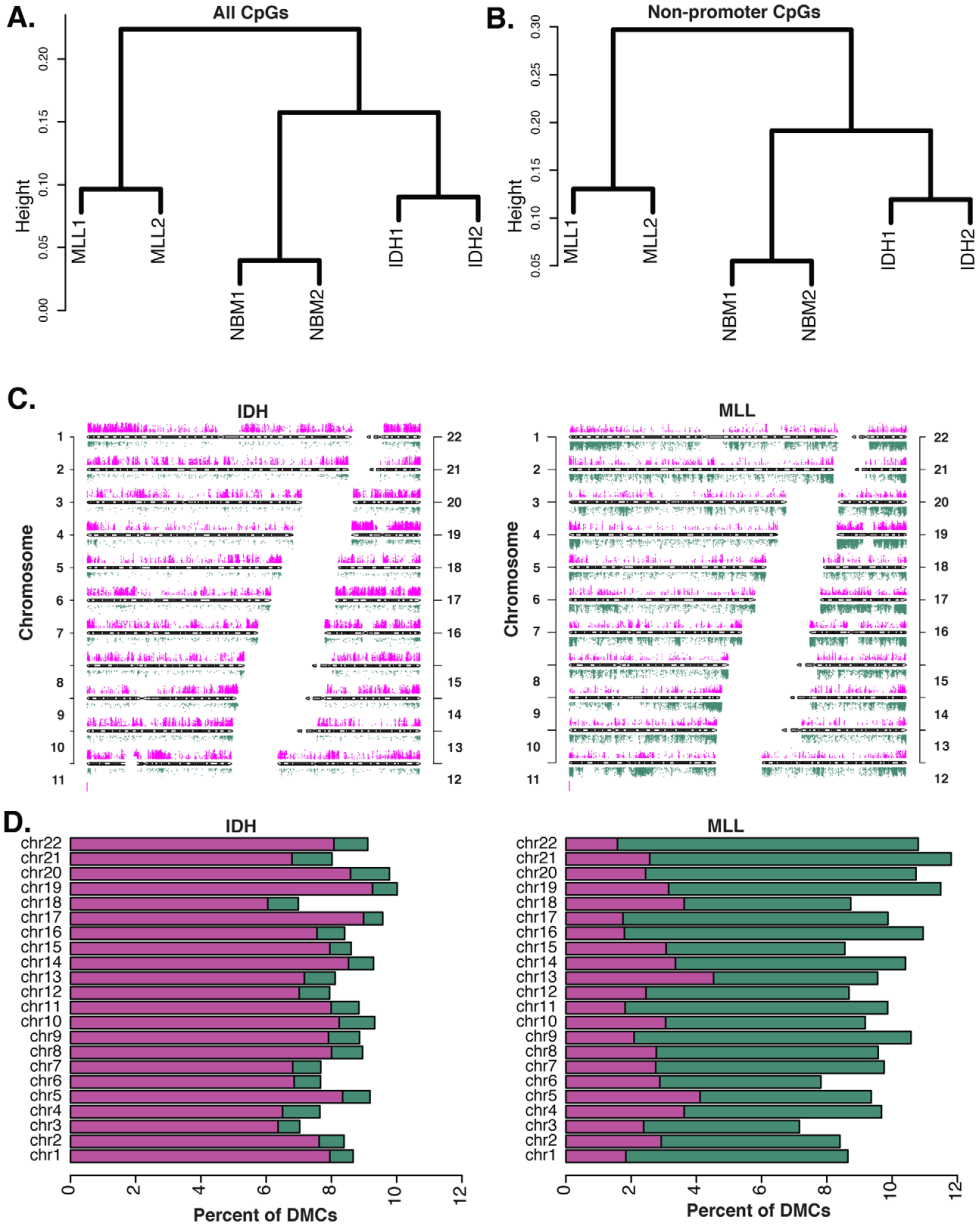
Diametrically opposed DNA methylation patterns in MLLr and IDH-mut AMLs. (A) Unsupervised analysis of DNA methylation by ERRBS using hierarchical clustering (distance = 1-Pearson correlation, Ward's agglomeration method) segregates the samples into their three biological groups using all CpGs. (B) This segregation is maintained when unsupervised analysis is performed on non-promoter CpGs. (C) Chromosome ideogram representing differential methylation in IDH-mut AMLs vs. NBM (left) and MLLr AMLs vs NBM (right). Only CpGs with q-value<0.01 and methylation difference of at least 25% are shown. Magenta points represent hypermethylation and green ones represent hypomethylation relative to NBM. (D) Stacking barplots showing percentage of hyper and hypomethylated DMCs out of all covered CpGs for each chromosome in IDH-mut AMLs (left) and MLLr AMLs (right). Green represents proportion of hypomethylated DMCs and magenta represents hypermethylated ones.

We previously reported that IDH-mut and MLLr AMLs distribute to different DNA methylation clusters and have distinct promoter DNA methylation signatures compared to normal CD34+ bone marrow controls (NBM) [10], [11]. We performed ERRBS in two IDH-mut AML samples, two MLLr cases harboring t(9;11)(q22,q23) translocations, and two NBM samples. ERRBS covered an average of 2,082,426 CpGs per sample. In addition to the expected high correlation between the NBMs (r = 0.96) there was a remarkable degree of correlation between the two IDH patients (r = 0.93) and the two MLLr patients (r = 0.92) (Figure S2), which far exceeded the correlation between MLLr and IDH-mut patients (r = 0.85–0.88), suggesting a strong link between genetic background and its effects on cytosine methylation. Unsupervised analyses using hierarchical clustering (1-Pearson correlation distance + Ward clustering method) and principal component analysis revealed that, even with this greatly enhanced representation of the genome, ERRBS methylation profiles from IDH-mut and MLLr naturally segregate from each other just as strongly as from NBM (Figure 2A and Figure S3A). In order to determine whether this natural segregation was driven solely by promoter differences in methylation, as captured in our previous studies, or whether biologically relevant differences were conserved in all genomic regions, we repeated the clustering analysis using only CpG sites within defined genomic regions. We found that using either (1) all non-promoter CpGs, (2) non-promoter intron CpGs, or (3) CpG sites at CpG islands and shores regardless of genomic location, the clustering results still retained the natural segregation into the biological groups (Figure 2B and Figure S3B–S3E). Notably, when the clustering was performed on CpG island-associated CpG sites (Figure S3D), IDH-mut AMLs segregated further apart from NBMs and MLLr AMLs, indicating that these sites are likely to be more heavily involved in the aberrant DNA methylation profiles of these AMLs. These findings demonstrate the existence of robust AML subtype-specific DNA methylation patterns, which extend beyond promoters to include other genomic regions.

### Diametrically opposed aberrant DNA methylation in IDH-mut and MLLr AML subtypes

In order to identify the nature of the differences between IDH-mut and MLLr AMLs , the cytosine methylation profiles of these tumors were compared to normal CD34+ bone marrow cells from healthy donors (NBM), using logistic regression (FDR at alpha = 0.01). In addition to statistical significance, we required a minimum cutoff of 25% methylation difference. This analysis revealed striking differences in the way that these two forms of AML differed from normal hematopoietic stem and progenitor cells. Specifically, we observed that IDH-mut AMLs display profound hypermethylation distributed across all chromosomes. In marked contrast, comparison of the cytosine methylation profiles of MLLr AMLs to NBM samples identified a predominance of aberrantly hypomethylated CpG site (Figure 2C and 2D). More specifically, we identified 62,367 differentially methylated cytosines (DMC) between IDH-mut AMLs and NBM, 89.6% of which were aberrantly hypermethylated in the leukemias and only 10.4% hypomethylated. Among the 85,216 DMCs identified in MLLr AMLs we observed a vastly different and opposing distribution (Chi-square test, p-value<0.0001), with only 28.5% of DMCs displaying hypermethylation and 71.5% being hypomethylated. The above results remained valid even when we used a more stringent cutoff of 40% methylation difference or a more relaxed cutoff of 10% (Figure S4A and S4B). These results demonstrate that the directionality of DNA methylation changes acquired during malignant transformation of myeloid hematopoietic cells is not uniform across all AML subtypes and that DNA methylation changes are indeed diametrically opposed in these two AML subtypes.

### Mutually exclusive targeting of aberrant CpG methylation sites in IDH versus MLL mutant AMLs

**Figure 3:**
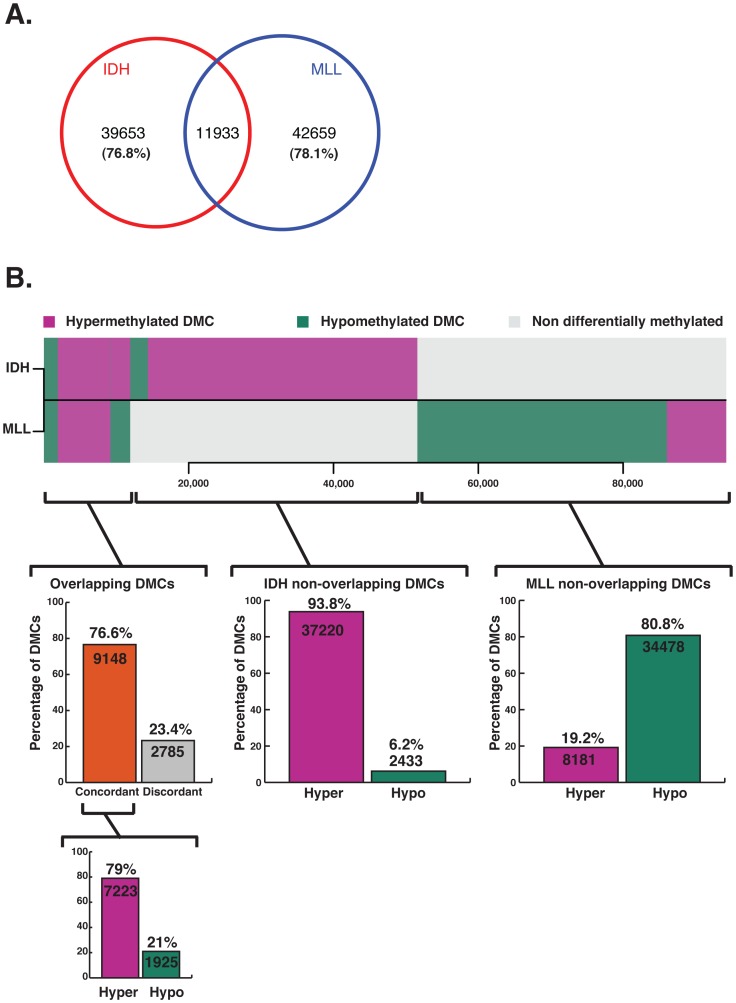
Aberrant methylation targets a minimally overlapping set of CpGs in IDH-mut and MLLr AMLs. (A) Venn diagram representing differentially methylated CpGs identified for IDH-mut and MLLr AMLs from amongst the 574,178 CpGs adequately represented (>10× coverage) across all six samples. Most DMCs are unique to either AML subtype, with minimal amount of events occurring at common sites across IDH-mut and MLLr. (B) Horizontal barplot comparing the methylation status of CpG sites at DMCs in IDH-mut AML (top) and MLLr AML (bottom). Magenta depicts >25% hypermethylation relative to NBM, green represents >25% hypomethylation and gray represents no differential methylation. Most DMCs are non-overlapping between the two subtypes of AML and display opposite changes in methylation. However, amongst the smaller set of DMCs that do overlap between the two AML subtypes, the vast majority (76.6%) are concordantly changed, with a clear predominance for aberrrant hypermethylation of those sites (79%).

Previous studies in AML were restricted to promoter microarrays [11], [25] or locus specific assays [25] that do not provide wide-spread and unbiased base pair resolution. Thus, it is not yet fully understood how aberrant DNA methylation is distributed in AML beyond these limited regions. Moreover, it is not clear whether results from studies carried out on certain solid tumor specimens [12], [13] are generally applicable to cancer, nor whether genetic background of tumors, and more specifically AML, can have an influence on what regions are perturbed. The base pair resolution and extended genomic coverage of ERRBS make it well suited to address these questions. To compare methylation status across all samples, we first identified a total of 574,178 CpGs adequately represented by ERRBS (>10× coverage; on average 53× coverage per base) in all specimens. Of these, 94,245 CpGs were differentially methylated (methylation difference >25%) in either one or both subtypes. Notably, 87.3% (n = 82,312) of these DMCs were non-overlapping and thus unique to either IDH-mut or MLLr leukemias (Figure 3). More specifically, 51,586 DMCs were identified in IDH-mut AMLs, of which the majority of CpGs, or 76.8%, were unique and non-overlapping with MLLr. In the case of MLLr AMLs, there were 54,592 DMCs, 78% of which were unique and non-overlapping with IDH-mut cases. Even more strikingly, 93% of the IDH-mut specific DMCs were hypermethylated vs. NBM, whereas 80.8% of MLLr specific DMCs were aberrantly hypomethylated. Comparable results were observed even when either a more stringent 40% or a less stringent 10% cutoff was used for calling DMCs (Figure S4). Pathway enrichment analysis of the DMCs observed in each subtype was performed using GREAT [26]. Only pathways with an FDR q-value<0.05 in both the hypergeometric and binomial tests were included. This analysis revealed that IDH-mut DMCs were enriched in several pathways, including cadherin, Notch and TGFb signaling (Table S3A). MLLr DMCs on the other hand featured enrichment of two pathways, one involving integrin signaling while the other included transcriptional activators *EP300, CREBBP, FOS, JUN* as well as several genes involved in regulation of apoptosis such as *BAX, CASP3, CASP6 and TP73* (Table S3B). Hence the DNA methylation defect of these two AML subtypes is not only perturbed in opposite directions but is also based on the differential methylation of an almost completely distinct set of CpGs, which affect distinct pathways.

Since our alignment strategy spanned the entire genome and used exact matches, we were able to determine whether DMCs were preferentially associated with certain repetitive sequences in the genome. Overall, we found that only 15% of CpG sites covered by ERRBS in all samples overlapped with repeat regions. However, we found that hypomethylated DMCs were enriched for repeat elements, with 24% overlap in MLLr (Odds-Ratio: 1.8, p-value 2.2e-16) and 26% in IDH-mut (Odds-Ratio: 2.0, p-value 2.2e-16), and most of those DMCs were found at Alu elements (8% in IDH-mut and 10% in MLLr). Hypermethylated DMCs, on the other hand were depleted for repeat elements, with only 7 and 8% of hypermethylated DMCs overlapping with repeats in IDH-mut and MLLr, respectively (Odds-Ratio for both 0.4, p-value 2.2e-16). These were, mostly low complexity and simple repeats (Figure S5).

Next we examined the common differentially methylated CpG sites in IDH-mut and MLLr AML (n = 11,933). Of these, 76.6% (n = 9148) were coordinately differentially methylated in the same direction in both AML subtypes, and the majority of these DMCs were aberrantly hypermethylated vs. NBMs (79%, n = 7223). These concordantly hypermethylated DMCs were more frequently associated with polycomb repressive marks than concordantly hypomethylated DMCs (66.2% vs. 46.2%, p-value<2.2e-16, Fisher's exact test). Concordantly hypermethylated CpGs were associated with genes involved in Cadherin, Wnt and Notch signaling pathways, many of which have been previously reported as frequently methylated in a variety of neoplasms, such as APC2 [27], [28], SFRP2 [29], CDH13 [30], [31], CDH15 [32] and PCDH10 [33], [34] (see Table S4). However, concordantly hypomethylated CpGs were not associated with any pathway but were instead significantly associated with repeat elements: 27.7% of concordantly hypomethylated CpGs overlapped with repeats, but only 7.4% of concordantly hypermethylated CpGs overlapped with a repeat (Fisher's exact test p-value<2.2e-16). This degree of overlap is similar to what we observed in the more global analysis of repeat elements mentioned above, indicating that concordantly hypomethylated DMCs are not enriched for repeat elements compared to subtype-specific DMCs. Hence, although the majority of DMCs in these two AML subtypes affect different CpGs in opposite directions there remains a core set of commonly affected CpGs, which are mostly concordantly hypermethylated regardless of genetic background. These results are consistent with an observation based on HELP assays indicating the frequent hypermethylation of a core set of 45 genes in AML [11]. Despite the differences in coverage between ERRBS and the microarray platform used in our previous studies, we found that 15/18 genes covered by both assays again presented with aberrant CpG hypermethylation in both AML subtypes in this current study (Table S5). Altogether, the data suggest two layers of epigenetic programming in AML, the first represented by perturbations specific to tumor subtype, and the second encompassing defects representative of the leukemic phenotype in general.

### Differential deregulation of CpG shores versus CpG islands according to genetic background

**Figure 4:**
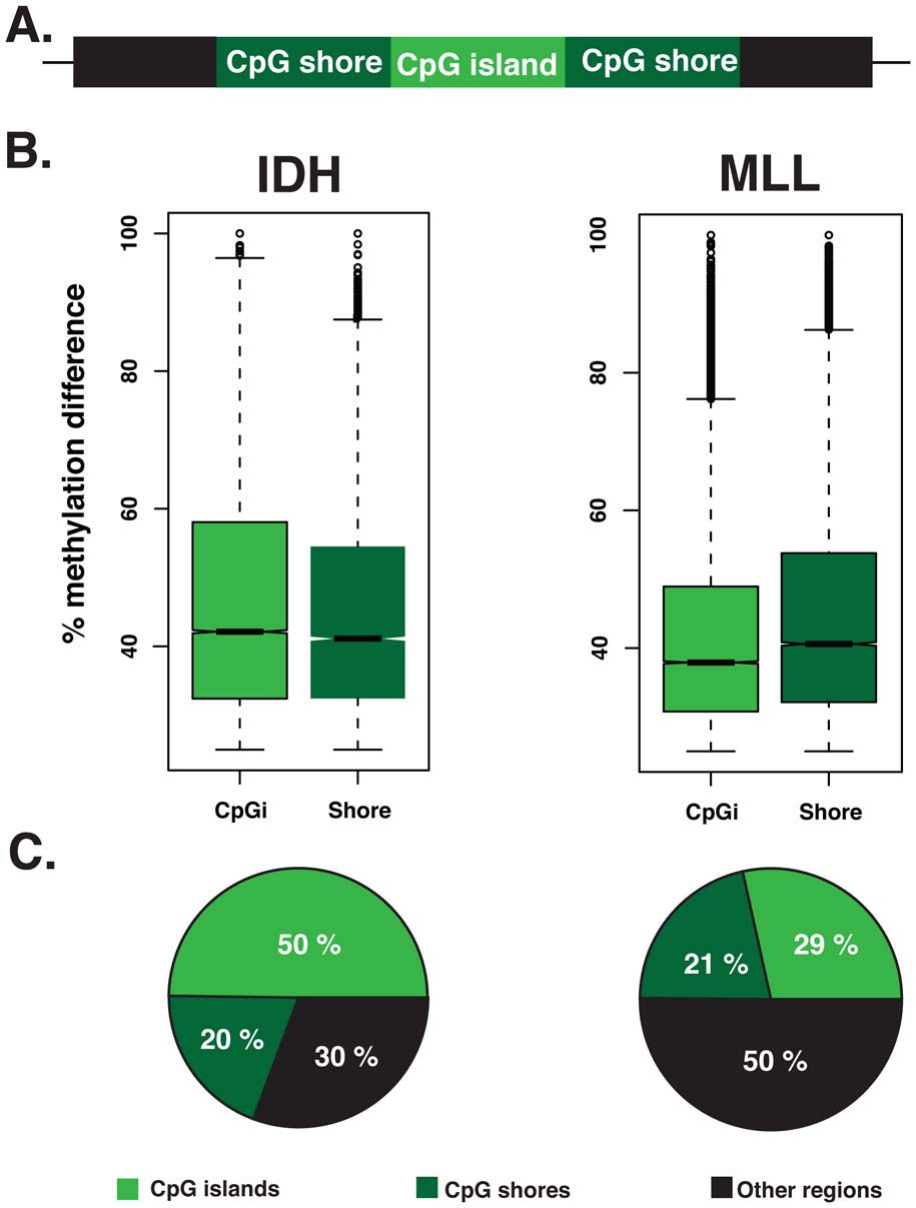
CpG islands and CpG shores show subtype-specific changes in the two types of AML. (A) Schematic representation of a CpG island (light green), flanked upstream and downstream by 2 kb CpG shores (dark green) and the region that extends beyond CpG shores (black). (B) Boxplots illustrating the magnitude of the methylation difference relative to NBM at DMCs that are annotated to CpG islands and CpG shores in either IDH-mut (Left) or MLLr (right) AMLs. (C) Pie charts illustrating the relative proportion of DMCs annotated to CpG islands (light green), CpG shores (dark green) and regions beyond CpG shores (black) in IDH-mut (left) and MLLr (right) AMLs.

Different types of analyses and platforms used in previous studies have tended to favor either aberrant methylation of CpG islands [14], [35] or CpG shores [12], [13] as the dominant defect in tumors. However it is not clear whether these observed differences are dependent on tumor type and/or methodology utilized in the different studies. The use of the ERRBS platform allowed us to explore differential methylation of both of these regions simultaneously. In order to understand which genomic regions present the highest variation in leukemias compared to NBM cells, we calculated differential methylation levels for individual CpG sites annotated to both CpG islands and shores. Our data revealed that CpG shores represented the regions with the highest variability in methylation in the MLLr AMLs (Wilcoxon rank sum test p-value 3.190e-11) (Figure 4B). In contrast, IDH-mut AMLs had higher variability in DNA methylation at CpG islands than CpG shores (Wilcoxon rank sum test P- value<2.2e-16).

We also observed significant differences in the absolute numbers of DMCs distributed to CpG islands and shores between the two subtypes (Chi-square test, p-value<0.0001). Specifically, we found that DMCs more frequently mapped to CpG islands in the IDH-mut AMLs cases (50% in IDH-mut vs. 29% in MLLr). In contrast, 50% of DMCs in the MLLr AMLs were found neither at CpG islands nor CpG shores but were instead annotated to regions even beyond CpG shores (Figure 4C). These findings indicate that distribution of DNA methylation changes during malignant transformation do not follow a uniform rule across all tumor types and genetic backgrounds, but rather that specific changes within and beyond genes are observed with distinct malignancy driving mechanisms.

### Distinct regional distribution of aberrant DNA methylation in IDH versus MLL AMLs

**Figure 5:**
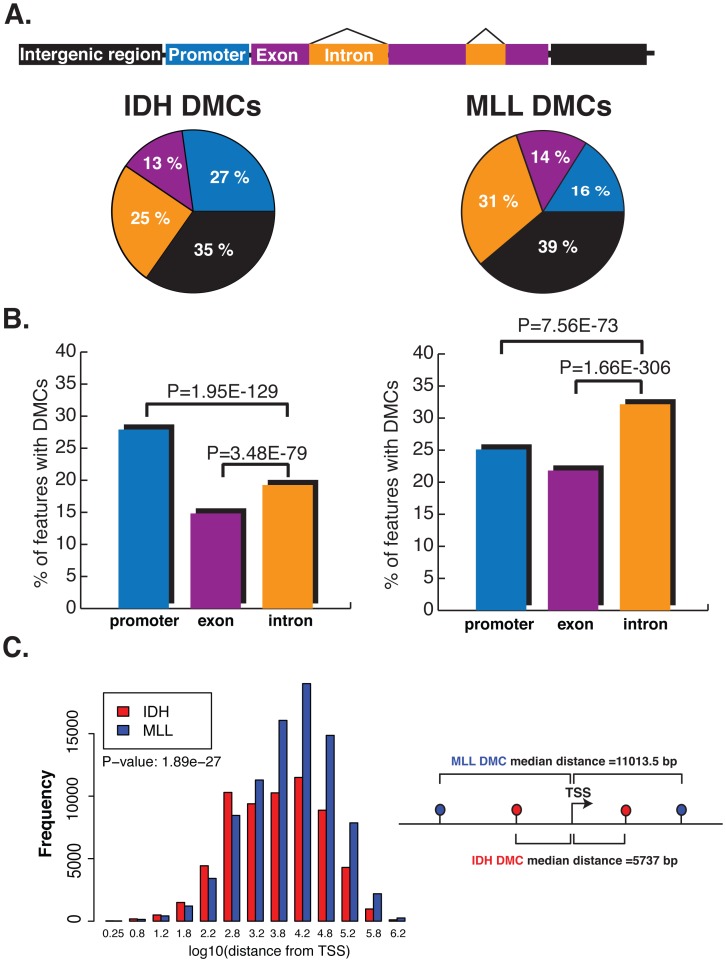
DMCs affect distinct genomic regions in IDH-mut and MLLr AMLs. (A) Top: cartoon representation of the different genomic regions analyzed. Bottom: Pie charts illustrating the proportions of DMCs annotated to promoter regions (blue), exons (magenta), introns (orange) and intergenic regions (black) in IDH-mut (left) and MLLr (right) AMLs. (B) Barplots representing the percentage of promoters, introns and exons overlapping with a DMC in IDH-mut (left) and MLLr (right) AMLs. Significantly higher proportion of promoter regions were overlapping with a DMC in IDH-mut over introns and exons, while introns were the most frequently affected regions in MLLr AMLs. (C) Histogram representing the log10 distance of DMCs to the nearest Transcription Start Site (TSS) in IDH-mut (red) and MLLr (blue) AMLs.

When considering the relation of DMCs to RefSeq annotated genes we observed that approximately 40% of all DMCs in both AML subtypes were found within gene bodies. However, more detailed analysis identified markedly dissimilar regional distribution of DMCs between the IDH-mut and MLLr AMLs. First, MLLr AMLs displayed significantly more abundant DMCs at introns than IDH-mut AMLs (31 vs. 25%) and intergenic regions (39 vs. 35%). In contrast, promoter-associated DMCs were twice as frequent in IDH-mut AMLs (27 vs. 16%) (Figure 5A) (Chi-square test, p-value<2.2e-16). A similar trend exists if we look at the percentages of introns, exons and promoters overlapping with a DMC in MLLr and IDH-mut. In IDH-mut, promoters more frequently overlap with DMCs whereas in MLLr, introns more frequently overlap with DMCs. This result demonstrates preferential localization of DMCs in different samples, where variability of methylation shifts its focus to different genomic features (Figure 5B). Moreover, the median upstream distance from the transcription start site (TSS) to observed DMCs was significantly greater in MLLr AMLs than in IDH-mut AMLs (11,013 bp vs. 5,737 bp, Wilcoxon rank sum test p-value<2.2e-16, Figure 5C). These analyses reveal yet another layer of difference between the two AML subtypes, with IDH-mut AMLs mainly affecting DNA methylation of CpG island promoter regions surrounding the TSS whereas MLLr AMLs mainly disrupt upstream and downstream regions, mostly independent of CpG islands. When considering promoters according to CpG frequency (as defined by Weber et al according to CpG ratio, GC content and length of CpG-rich region [36]), we found that more of the high CpG promoters (HCPs) overlap with DMCs in both IDH-mut and MLLr compared to low CpG promoters (LCPs) (17.1% vs 7.9% in IDH-mut: p-value<2.2e-16 and, 11.1% vs 4.9% in MLLr: p-value = 9.4e-12). However, it was intermediate CpG promoters (ICPs) that were the most enriched with DMCs in both leukemia subtypes: 73% of ICPs in IDH-mut and 71% of ICPs in MLLr with covered CpGs overlapped with DMCs.

We then examined these regional differences in cytosine methylation relative to known regulatory elements. We compared the DMC sites from both IDH-mut and MLLr AMLs to available ENCODE data sets [37] for CTCF binding and H3K4me1 and H3K4me3 data to define enhancer sites (defined as sites positive for H3K4me1 and devoid of H3K4me3) [38]. We found that CTCF binding sites and enhancers were more frequently found in the vicinity of MLLr DMCs (+/−500 bp) than of IDH-mut DMCs (Fisher's exact test p-value: <0.001 for both CTCF and enhancer sites). Enhancers and CTCF binding sites were more frequently hypomethylated in MLLr AMLs (Fisher's exact test p-value<0.001), while in IDH-mut AMLs these sites were more frequently hypermethylated (Fisher's exact test p-value<0.001) (Table 1). Whereas the mechanism through which IDH mutations affect particular genes is unknown, MLL fusion proteins are known to directly upregulate specific target genes, such as HOXA9, which are essential for the transformation process [2]. To investigate this, we surveyed the genomic localization of MLL fusions, MEIS1 or HOXA9 by ChIP-seq and examined whether aberrant DNA methylation was associated with binding of these factors. We found that MLL bound more frequently at promoters, and that only 6.5% of the 833 MLL peaks covered by the ERRBS assay occurred within 500 bp of MLLr DMCs. While only 49 out of 614 of the HOXA9/MEIS1 peaks [39] were covered by the ERRBS assay, 24.4% of them were associated with DMCs in MLLr AMLs, the majority of which were hypomethylated (22.4% vs 2%, Fisher's exact test p-value<0.004), suggesting that aberrant hypomethylation in MLLr AMLs is more closely linked to the HOXA9 and its co-factor MEIS1 than to the MLL fusion protein itself.

**Table 1:** Overlap of differentially methylated cytosines with CTCF, MLL and HOXA9 binding sites, and enhancer regions.

MLLr	any Hoxa9	any Meis1	Hoxa9 and Meis1	Hoxa9 or Meis1	MLL	CTCF	Enhancers
Peaks with Hypomethylated DMCs	5(19.2%)	9(23.6%)	3(20%)	11(22.4%)	36(4.3%)	6865(31.9%)	17646(35.7%)
Peaks with Hypermethylated DMCs	1(3.8%)	1(2.6%)	1(6.6%)	1(2.0%)	19(2.2%)	2150(10.0%)	3268(6.6%)
Peaks with read coverage	26	38	15	49	833	21474	49321
p-value hypo vs. hyper peaks proportion (Fisher's)	0.19	0.01	0.6	0.004	0.03	<2.2e-16	<2.2e-16
Peaks with DMCs	6(23.0%)	10(25.2%)	4(26.6%)	12(24.4%)	55(6.5%)	9025(41.9%)	20914(42.3%)

IDH-mut	any Hoxa9	any Meis1	Hoxa9 and Meis1	Hoxa9 or Meis1	MLL	CTCF	Enhancers
Peaks with Hypomethylated DMCs	2(7.1%)	2(5.2%)	1(6.6%)	3(5.8%)	12(1.4%)	1055(4.6%)	2778(5.1%)
Peaks with Hypermethylated DMCs	3(10.7%)	2(5.2%)	1(6.6%)	4(7.8%)	88(10.7%)	5840(25.7%)	9282(17.0%)
Peaks with read coverage	28	38	15	51	818	22653	54322
p-value hypo vs. hyper peaks proportion (Fisher's)	1	1	1	1	2.97E-16	<2.2e-16	<2.2e-16
Peaks with DMCs	5(17.8%)	4(10.4%)	2(13.2%)	7(13.6%)	100(12.3%)	6895(30.3%)	12060(22.1%)
p-value between subtypes (comparisons of features with DMCs in subtypes)	0.74	0.13	0.65	0.21	9.58E-05	7.33E-142	0
total number of Peaks	303	458	147	614	1108	101102	416282

### Context-dependent association of DNA methylation with gene expression in AMLs

**Figure 6:**
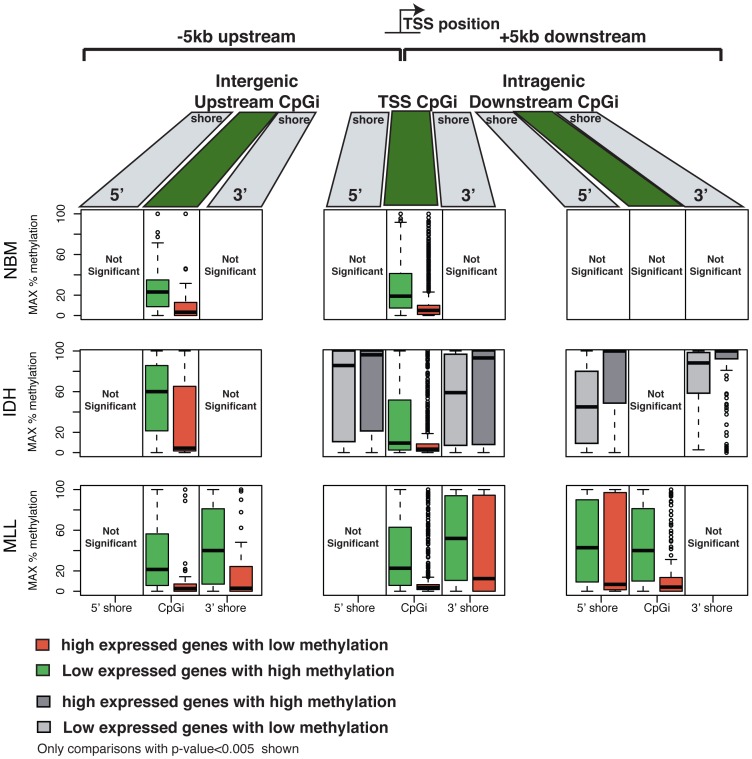
DNA methylation and gene expression relationships display subtype-specific differences. CpG islands and shores across the genome were categorized into those located upstream from a transcription start site (TSS), overlapping a TSS or located downstream from a TSS. Boxplots are plotted that illustrate the maximum DNA methylation levels at CpGs within these CpG islands and CpG shores for the top 15th percentile expressed genes (right) and the bottom 15th percentile expressed genes (left). Each row shows a representative sample for each type: Normal bone marrow (top); IDH-mut AML (middle) and MLLr AML (bottom). In all sample types CpG islands overlapping a TSS displayed lower methylation levels in highly expressed genes and higher methylation levels in genes that were expressed at low levels. In MLLr AMLs this relationship between expression and methylation levels further extended into CpG shores, and was also observed at CpG islands and shores upstream and downstream from the TSS. IDH-mut AMLs, and to a lesser degree NBM samples, displayed higher methylation levels at CpG shores of genes with high expression levels, while low methylation levels were observed at these shores for genes expressed at low levels.

In order to determine the potential functional significance of the distinct DNA methylation patterning observed in IDH-mut and MLLr AMLs we examined gene expression microarray profiles from the same AML cases [10], [40]. We assigned CpG sites into 3 types of regions: CpG islands overlapping a TSS, intergenic CpG islands upstream of the TSS (up to −5 kb) and intragenic CpG islands downstream of the TSS (up to +5 kb). Both in normal CD34+ samples and leukemia specimens, hypomethylation within CpG islands overlapping TSSs was associated with highly expressed genes, while hypermethylation was observed for low expression genes (top and bottom 15^th^ percentile of expressed transcripts, Wilcoxon rank sum test p-value<0.005) (Figure 6 and Figure S6). However, the relationship between CpG shore methylation status and gene expression levels was different in all three sample types. Hypermethylation of CpG shores was associated with low levels of gene expression only in MLLr AMLs, both at CpG shores overlapping the TSS as well as at downstream intragenic and upstream intergenic CpG shores. In marked contrast, CpG shore methylation levels in IDH-mut AMLs behaved in the opposite way, so that lower levels of methylation were in fact associated with lower levels of expression (Wilcoxon rank sum test p-value<0.005), while in normal CD34+ cases gene expression levels did not appear to depend on CpG shore methylation status at all.

Furthermore, when examining DMCs and their correlation with differential gene expression between the different AMLs and the normal bone marrow specimens, we found that only DMCs at the core promoter regions were significantly inversely correlated with differential gene expression in IDH-mut AMLs (p-value = 0.0047). However, for MLLr AMLs, we observed that while core promoter DMCs were also significantly associated with differential expression (p-value = 3.1e-16), this association was also significant at upstream DMCs (up to 10 kb), both for DMCs located at CpG islands (p-value = 3.2e-11) and CpG shores (p-value = 1.3e-23). Finally, downstream intronic DMCs overlapping with CpG islands also showed a significant correlation with expression in MLLr AMLs (p-value = 0.046). Collectively, these findings suggest that subtype specific DNA methylation distribution in AMLs regulates gene expression in a subtype-defined manner. More precisely our data indicate a significant role for long-range epigenetic regulation in MLLr AML through distal intergenic and intronic CpG islands, whereas IDH-mut AMLs display a predominance of promoter-centric epigenetic regulatory effects.

## Discussion

The study of gene promoters and CpG islands under the assumption that variation in the 5-methylcytosine status at these locations would have functional importance has been the focus of most cancer-related DNA methylation studies. Building upon the previously described RRBS method, the ERRBS assay described here made it possible to measure DNA methylation in primary AML samples beyond promoter regions, extending even into distal intergenic regions. This significantly enhanced genomic coverage allowed us to demonstrate that heterogeneity in epigenomic profiles in AML is not only a factor of different genes being affected, but rather encompasses a far more complex scenario, which includes the aberrant DNA methylation of distinct regions of the genome as well as differential mechanisms of regulation of gene expression according to genetic background. Given the specificity and reproducibility of these aberrant DNA methylation patterns, it is likely that their establishment in malignant cells is directly linked to genetic driver lesions. Our previous studies using HELP promoter microarrays are consistent with these results in that they revealed a hypermethylated promoter signature in IDH-mut AMLs, and a hypomethylated signature in MLLr. However those studies did not have the resolution or depth to reveal the true genomic nature, complexity and qualitative differences that we are now able to report regarding the nature of cytosine methylation distribution in these AML patients.

Specifically, in the case of MLLr leukemias, aberrant DNA methylation consists mostly of aberrant hypomethylation of upstream and intronic CpGs including CpG islands and shores, but extending to and heavily involving even more distal regions. Hypomethylation of regulatory elements is consistent with the actions of MLL fusion proteins as transcriptional activators. However, in these tumors the distal localization of hypomethylation was more closely associated with the presence of HOXA9 and MEIS1 binding sites and enhancer regions than with MLL binding sites, suggesting that aberrant DNA hypomethylation in these tumors may be more closely related to effects of downstream targets of MLL than to the fusion protein itself. However, it should be noted that a subset of MLL peaks (6.5%) did indeed overlap with DMCs in the MLLr AMLs. Since our ChIP-seq antibody recognized both the wild-type and the rearranged copy of MLL. Given that MLL fusions have been shown to bind to a subset of wt-MLL target genes [41], it still remains possible that the subset of overlapping peaks may be preferentially bound by the MLL fusion. Further studies with antibodies capable of distinguishing between the two forms of MLL will be required to properly address the role of MLL fusions in helping establish the aberrant methylation profile seen in these leukemias. The functional relevance of hypomethylation in MLLs is supported by the enrichment for highly transcribed genes at loci where this distal methylation pattern is observed. IDH mutant AMLs on the other hand, display a diametrically opposed pattern of aberrant methylation of CpGs, which results in the prefential hypermethylation of CpG islands surrounding TSSs, involving an almost entirely mutually exclusive set of CpGs but also resulting in the downregulation of genes with increased methylation. While it is clear that the generation of the 2-HG metabolite by the mutant forms of IDH1 and IDH2 results in inhibition of the hydroxylation reaction by TET proteins [6], it is as yet unclear why this inhibition results in a promoter-specific hypermethylation pattern, and inhibition of other epigenetic modifiers such as Jumonji C domain histone demethylases by 2-HG [42] may also play a role in determining the aberrant epigenetic profiles of these AMLs. Moreover, it is possible that hydroxymethylation of DNA by TET proteins may depend on other DNA binding partners that direct them to specific genomic sites.

Even though the two AML subtypes were dramatically different, they still shared a core hypermethylated signature including genes previously shown to be almost universally hypermethylated in AMLs [11]. Similar to what had been previously demonstrated in colon cancer murine models [43], [44], Broske and colleagues demonstrated that DNA methylation is required to fully transform hematopoietic stem and progenitors, even with a potent oncogene such as MLL-AF9 [45]. Taken together, these observations point towards the existence of a core of hypermethylation lesions that are a necessary event during malignant transformation, and that likely cooperate with the underlying genetic events in the different AML subtypes.

Most importantly, abnormal DNA methylation patterning does not occur in a stereotypical manner in cancer, but instead adopts distinct and specific distributions dependent at least in part on genetic background, even when comparing cases of the same tumor type with different driver mutations. Our analysis comparing gene expression and DNA methylation at base-pair resolution across three different sample types demonstrates that epigenetic regulation of gene expression in tumors may at least in part be context dependent, suggesting that cell-type specific factors may come into play to establish and maintain unique regulatory mechanisms in these cells. Finally, the large distances between DMCs and transcription start sites support a potential role for epigenome regulation at distal regulatory elements, via looping or other mechanisms, in directly influencing the specificity of the transcriptional machinery. Taken together our data support the existence of divergent roles of the epigenome in regulating the transcriptional profiles of AML and indicate that altered gene expression is associated with the differential methylation of distinct and non-overlapping CpGs and regions in tumors with different genetic backgrounds. Moreover, in the case of MLLr AMLs, these abnormal regulatory mechanisms extend far beyond the classically described cancer-associated promoter CpG island hypermethylation, and indicate that distal intergenic DNA methylation abnormalities may also have functional consequences in certain tumors. These findings are consistent with those described by other groups which have seen an association between differentially methylated regions at CpG shores in solid tumors and changes in gene expression [13]. Indeed, these significant regional and CpG specific differences would be unlikely to be captured with any other method except whole genome bisulfite sequencing or methods like ERRBS with unbiased and adequate base-pair resolution detection of CpG methylation. Of note, the gene expression microarrays used in the current study only capture known coding transcripts. Yet the expanded coverage of ERRBS can also provide information on putative regulatory elements of non-coding RNAs as well as information on regulation of alternative promoters. It will be important for future studies perhaps using RNA-seq, to analyze the relationship between aberrant DNA methylation and the expression levels of non-coding RNAs or, the correlation between DNA methylation status at alternative promoters and the expression levels of transcript variants, a regulatory role previously described for DNA methylation [46]. High resolution comparative studies of genetically characterized primary human tumors using methods that adequately represent the genome at base pair resolution (such as RNA-Seq) may thus yield a wealth of new information on mechanisms driving tumor transcriptional and epigenetic programming and the true scope and nature of aberrant DNA methylation patterning in cancer. Studies integrating more comprehensive transcriptome data with transcription factor binding and histone modification patterns in concert with assays designed to explore chromosomal structure will yield further insight into such mechanisms.

## Methods

### Cell lines

The human colorectal carcinoma cell line HCT116 was a kind gift from Dr. John Mariadason. The cell line was maintained in DMEM supplemented with 10% fetal bovine serum (FBS), 100 units/ml of penicillin and 100 µg/ml of streptomycin (Invitrogen) at 37°C and 5% CO_2_. The HCT116 DNMT1(−/−) DNMT3b(−/−) double knockout clone number 2 (DKO) cell line was a kind gift from Dr. Steve Baylin. The cell line was grown in McCoys'5A medium with 10% FBS, 0.2 mg/ml Neomycin(G418), and 0.1 mg/ml Hygromycin B. Genomic DNA was extracted from the cell lines using standard phenol:chloroform extraction followed by ethanol precipitation.

### Primary samples

AML samples were obtained from previously reported, de-identified patient samples, from individuals enrolled in the Eastern Cooperative Oncology Group's (ECOG) E1900 clinical trial [47] and from patients seen at Erasmus University MC, The Netherlands. Two IDH1/2 mutant AML samples (IDH1 and IDH2), two mixed lineage leukemia gene rearranged AML samples harboring t(9;11) (MLL1 and MLL2) and one additional AML sample (AML) were available for processing. Two normal CD34+ bone marrow control samples were purchased from AllCells, LLC (Emeryville, CA, USA). Institutional review board approval was obtained at Weill Cornell Medical Center and this study was performed in accordance with the Helsinki protocols. DNA was isolated from each primary sample using the Qiagen Puregene kit per manufacturer's recommendation.

### Reduced representation bisulfite sequencing (RRBS)

RRBS was performed as follows: i) 5, 50 or 1000 ng of high quality genomic DNA were digested with 200 U of MspI (New England Biolabs, NEB) which cuts DNA regardless of cytosine methylation status at CCGG sequence in a 100 µl reaction for up to 16 hours at 37°C. DNA was isolated using standard phenol chloroform extraction and ethanol precipitation and resuspended into 30 µl of 10 mM TrIs pH 8.0.ii) End repair of digested DNA was performed in a 100 µl reaction using 15 U of T4 DNA polymerase (NEB M0203L), 5 U of Klenow DNA polymerase (NEB M0210L), 50 U of T4 Polynucleotide Kinase (NEB M0201L), 4 µl of premixed nucleotide triphosphates each at 10 mM (NEB N0447L) using T4 DNA ligase buffer with 10 mM dATP (NEB B0202S). The reaction was incubated at 20°C for 30 minutes and products were isolated using QIAquick PCR purification columns per manufacturer's recommended protocol (Qiagen) into 32 µl of EB buffer. iii) Adenylation was performed in a 50 µl reaction using 15 U Klenow fragment (3′ to 5′ exo minus, NEB M0212L), 10 µl of dATP at 1 mM concentration using Klenow buffer (NEB). The reaction was incubated at 37°C for 30 minutes and products were isolated using MinElute PCR purification columns per manufacturer's recommended protocol (Qiagen) into 10 µl EB buffer. iv) Adenylated DNA fragments were ligated with pre-annealed 5-methylcytosine-containing Illumina adapters in a 20 or 50 µl reaction for 5 ng or 50 ng or higher starting materials respectively using 2000 U T4 DNA ligase (NEB M0202T) and 1.2 µM final concentration of methylated adapters at 16°C for a minimum of 16 hours. Products were isolated using MinElute columns per manufacturer's recommended protocol (Qiagen) into 10 µl EB buffer. v) Library fragments of 150–175 and 175–225 bp were gel isolated from a 1.5% agarose gel (using low range ultra agarose from Biorad) using the Qiaquick Gel Extraction kit per manufacturer's recommended protocol (Qiagen) into 40 µl EB buffer. vi) bisulfite treatment was performed using the EZ DNA Methylation Kit (Zymo Research) per manufacturer's recommended protocol with the following modifications: 1) incubation after the addition of CT conversion reagent was conducted in a thermocycler (Mastercycler ep gradient, Eppendorf) with the following conditions: 30 seconds at 95°C followed by 15 minutes at 50°C for 55 cycles and, 2) products were eluted into 40 µl nuclease free water. vii) PCR amplification for each library was performed in a 200 µl reaction containing 2 µl FastStart Hifidelity DNA Polymerase (Roche), 0.5 µM each of Illumina PCR primers PE1.0 and 2.0, 0.25 mM each nucleotide triphosphate using buffer 2 per manufacturer's recommendation and divided into four 50 µl reactions. The thermocycler conditions were: 5 minutes at 94°C, 18 cycles of 20 seconds at 94°C, 30 seconds at 65°C, 1 minutes at 72, followed by 3 minutes at 72°C. PCR products were isolated using AMPure XP beads per manufacturer's recommended protocol (Agencort) into 50 µl of EB buffer. viii) All amplified libraries underwent quality control steps including using Qubit 1.0 fluorometer and a Quant-iT dsDNA HS Assay Kit for quantitation (Invitrogen) and bioanalyzer visualization (Agilent 2100 Bioanalyzer).

Extended Reduced Representation Bisulfite Sequencing (ERRBS) was performed as described above for RRBS, except that in step number v, library fragment lengths of 150–250 bp and 250–400 bp were gel isolated.

#### Data deposition statement

All data have been deposited for public access in the GEO database. The Accession number is GSE37454.

### Sequencing

The amplified libraries were sequenced on an Illumina Genome Analyzer II or HiSeq2000 per manufacturer's recommended protocol for 50 bp single end read runs. Image capture, analysis and base calling was performed using Illumina's CASAVA 1.7.

### Quantitative DNA methylation sequencing by MassARRAY EpiTYPER

Validation of select CpG methylation in HCT116 cell line was implemented by MALDI-TOF mass spectrometry using EpiTYPER by MassARRAY (Sequenom, San Diego, CA) as previously described [48]. Primers were designed to cover CpGs in various chromosomal locations with various methylation levels and sequencing coverage. Primers and amplicon sequences are listed in Table S2.

### Computational approaches

#### Bisulfite treated read alignment and methylation calls

Reads were filtered from the adapter sequences using FAR software (Dodt, M, Ahmed R, Dieterich C. FAR – flexible adapter remover. FAR project website (2011) (http://sourceforge.net/projects/theflexibleadap/). Adapter sequence contamination usually occurs towards 3′ends of some reads. The adapter matching part of the read was removed if it aligned with the adapter sequence at least 6 base-pairs and had at most 0.2 mismatch error rate. Reads were aligned to whole genome using the bismark alignment software [24] with a maximum of 2 mismatches in a directional manner and only uniquely aligning reads were retained. In order to call methylation score for a base position, we required that read bases aligning to that position have at least 20 phred quality score and the base position should have at least 10× coverage. Only CpG dinucleotides that satisfy these coverage and quality criteria were retained for subsequent analysis. Percentage of bisulfite converted Cs (representing unmethylated Cs) and non-converted Cs (representing methylated Cs) were recorded for each C position in a CpG context.

#### Comparison of whole-genome alignment pipeline to MspI fragment pipeline

In silico MspI fragment library was constructed by cutting the reference genome to fragments from MspI sites. Bisulfite converted reads are aligned to in silico bisulfite converted MspI fragments using ELAND aligner. Similar to the whole genome alignment pipeline, in order to call methylation percentage score for a base position, we required that read bases aligning to that position have at least 20 phred quality score and the base position should have at least 10× coverage. We aligned reads from 4 samples to MspI fragments and whole genome and compared their alignment rate and number of covered CpGs.

#### CpG dinucleotide annotation

CpG islands, refseq genes and repeat sequences were downloaded from the UCSC genome browser [49]. CpG shores were defined as 2000 bp flanking regions on upstream and downstream of a given CpG island [13]. If a 2000 bp shore overlapped with another island, then the shore was clipped so that its last base falls before the start of the overlapping CpG island. Similarly, if shores were overlapping they were merged into a single shore. In addition, the genome was partitioned to intergenic, intron, exon and promoter regions. Promoter regions were defined as the 2 kb window centered on the transcription start sites (TSS) of refseq genes. We classified CpG dinucleotides as promoter, intronic, exonic or intergenic based on their overlap with these predefined regions. In addition, we classified CpG dinucleotides as CpG island or shore overlapping.

#### Methylation comparison and differential methylation

Percent methylation values for CpG dinucleotides are calculated by dividing number of methylated Cs by total coverage on that base. We clustered samples and calculated methylation correlations by comparing percent methylation scores of CpG dinucleotides that are covered across all samples (IDH-mut, MLLr and NBM samples).

Hierarchical clustering of the six samples was performed using the hclust function in R-2.14.0 (http://www.r-project.org/) where we used 1-Pearson correlation distance and Ward's agglomeration method.

Methylation values for genomic regions (intergenic, intron, exon and promoters, CpG islands and island shores) between different samples were compared by taking the mean methylation percentage of CpG dinucleotides overlapping those regions. In order to calculate the correlation between different samples and generate the appropriate scatter plots we required that in any given region at least 3 CpG dinucleotides were covered by reads in both samples.

The test for differential methylation was performed at the single base level. The test is performed only on CpG dinucleotides covered in all the test and control samples in each case. In order to improve the number of covered CpG dinucleotides across samples, we merged the read coverage on forward and reverse strand of a given CpG dinucleotide before doing the test. For the test, the number of methylated and unmethylated Cs aligning to each base were counted and compared across samples. To determine significant differential methylation between two sets of samples, we applied logistic regression and the likelihood ratio test. Observed p-values were adjusted with the SLIM method [50] We also calculated the percent methylation difference between the sets of test and control samples. We calculated the percent methylation values per sample set by adding up the methylated C counts for samples in the same set and dividing them by total read coverage of two samples on that base. Consequently, we subtracted these set specific percent methylation values from each other to get percent methylation difference between the sample sets.

### Pathway enrichment analysis

Pathway enrichment analysis was performed using the GREAT software [51], which associates genomic regions with nearby genes and calculates enrichment statistics using annotations of those genes. In order to associate genomic regions to genes, each gene is assigned to a regulatory domain, which consists of a basal promoter and extension around that promoter to cover distal elements. Following that, the genomic regions falling on those regulatory domains are associated with the genes. Following parameters are used for definition of regulatory domain: 5000 bp upstream, 1000 bp downstream of TSS as basal regulatory domain and this is extended up to 100 kb maximum. GREAT calculates two enrichment statistics using the binomial test and the hypergeometric test. Only the pathways significant by both tests are shown (FDR q-value<0.05).

### Gene expression relationship with methylation

Gene expression for IDH mutants and normal bone marrow cells are downloaded from the Gene Expression Omnibus (GEO) (accession: GSE24505). Normal bone marrow samples are not matched to the samples on this array however we averaged 5 normal bone marrow samples on the array to interpolate the expression profiles of our normal bone marrow samples. The sample matched gene expression profiles for cells with MLL translocation are downloaded from GEO (accession: GSE6891). Expression percentiles of each transcript are also calculated using R function “ecdf”. The transcripts for each sample are divided into two categories high expressed (the top 15%) and low expressed (the bottom 15%).

CpG islands are mapped to annotated transcripts for probes as follows. First, we mapped CpG islands to 10 kb window around the TSS of the annotated transcript, and CpG islands in this window are classified as TSS overlapping, upstream and downstream CpG islands depending on whether or not they overlap with TSS and relative location if they are not overlapping with TSS. Following that, we compared maximum methylation per island and maximum methylation per shore for high and low expressed genes on each sample. We used Wilcoxon's Rank sum test to compare maximum methylation distributions on each shore and CpG island for high and low expressed genes. For this comparison we only considered CpG islands and shores that have at least three genomic CpGs covered by bisulfite reads.

When correlating DMCs with the differential expression, we first calculated fold-change of MLLr vs. NBM and IDH-mut vs. NBM samples. Expression data for NBM samples (although not sample matched) were available for both IDH-mut and MLLr fold-change calculations within the respective microarray types and downloaded from GEO (accession numbers GSE24505 and GSE6891 respectively). We calculated fold-change between the average expression values of the groups. Following that we measured correlation between percent methylation difference at DMCs and fold-change of the nearest gene (obtained by extracting the nearest TSS) using “correlation.test” in R. We performed separate correlation analyses for DMCs at the core promoter (−300 bp,+300 bp around TSS), upstream from the TSS (up to 10 kb), within CpG islands (up to 5 kb from TSS), within CpG island shores (up to 5 kb from TSS), within intronic regions, at intronic CpGs, and at CpGs within intronic CpG islands and shores.

### Chromatin immunoprecipiation–sequencing of MLL and HOXA9

MLL ChIP-seq experiments were performed in the MLL-AF4 cell line RS4;11 (ATCC#CRL-1873) using antibodies to MLL1 (Bethyl Laboratories A300-086A). ChIP-seq libraries were prepared from 10 ng of immunoprecipitated material using Illumina's ChIP-seq kit as per manufacturer's instructions, and then sequenced on a Genome Analyzer IIx sequencer. Alignment against the human genome, peak calling and downstream analysis was performed using ChIP-seeqer [52]. HoxA9 and Meis1 ChIP-seq peaks from murine cells from Huang et al [39] were annotated to the human genome using the LiftOver function from the UCSC browser [49].

### ChIP–seq peak overlap with DMCs

The ENCODE CTCF, H3K27me3, H3K4me1 and H3K4me3 peak locations are downloaded using UCSC table browser [53]. ChIP-seq experiments and peak finding were carried out by The Broad Institute for 9 different cell lines only 8 of which had H3K4me1 and H3K27me3 marks available for download [54]. Polycomb repressive marks were identified as those with K3K27me3 by Ernst et al using a hidden-markov model based approach [54]. For enhancer markers, we picked H3K4me1 sites that do not overlap with H3K4me3 in a given a given cell line as previously shown [38]. We merged all such H3K4me1 sites from 8 cell lines, so that if H3K4me1 sites overlap in different cell lines they will not be counted twice. The same merging procedure is applied for CTCF binding sites and H3K27me3 from 8 cell lines. Following that, we extended the peak locations for CTCF, enhancer markers, MLL, Meis1 and HoxA9 by 500 bp on each side of the peak location. We overlapped resulting regions with DMCs in IDH-mut and MLLr. We also overlapped those regions with CpGs covered by reads to see how many of those binding sites are covered by ERRBS. We applied Fisher's exact test to compare proportions of DMCs.

## Supporting Information

Figure S1 ERRBS is highly reproducible and sensitive. (A) Correlation between CpG dinucleotides, CpG islands and promoter methylation levels using pearson correlation between technical replicas of ERRBS using 5, 50 or 1000 ng genomic DNA from the HCT116 cell line. (B) Distribution histograms of CpG coverage and CpG methylation levels along forward and reverse strands in HCT116 ERRBS results. (C) Distribution histogram of CpG methylation levels along forward and reverse strands in DKO ERRBS results. Similar results were obtained from reverse strand (data not shown) and CpG coverage distributions over both strands were similar to coverage seen with HCT116 sequencing (data not shown). (D) Technical validation of ERRBS performance in HCT116 at select CpGs by MassARRAY. Dot plot shows correlation between DNA methylation as measured by ERRBS (x-axis) and percent methylation as measured by MassARRAY EpiTyper (y-axis). (Correlation coefficient: 0.97).
(TIF)

Figure S2 Biological replica reproducibility. (A) Correlation plot of CpG dinucleotide methylation levels between two biological replica of ERRBS data using normal bone marrow controls (NBM_#1 and NBM_#2_Rep#2). (B) Correlation plot of CpG dinucleotide methylation levels between two biological replica of ERRBS data using IDH mutant AML samples (IDH-mut_#1 and IDH-mut_#2). (C) Correlation plot of CpG dinucleotide methylation levels between two biological replicas of ERRBS data using MLL translocated AML samples (MLLr_#1_Rep#2 and MLLr_#2).
(TIF)

Figure S3 DNA methylation patterns naturally segregate AML and NBM samples. Unsupervised analysis using either principal component analysis or hierarchical clustering (1-Pearson correlation distance + Ward's agglomerative algorithm) of DNA methylation by ERRBS at (A) all CpGs, (B) non-promoter CpGs, (C) non-promoter intron CpGs, (D) CpGs within CpG islands and (E) CpGs within CpG shores, segregates the samples into their three biological groups.
(TIF)

Figure S4 Differential methylation in MLLr and IDH-mut AMLs are preserved at 40% and 10% cutoffs. Chromosome ideogram representing differential methylation in IDH-mut AMLs vs. NBM (A) and MLLr AMLs vs. NBM (B), using changes greater than 10%. Light and dark magenta points represent hypermethylation changes relative to NBM of 10–40% and greater than 40% respectively. Light and dark green points represent hypomethylation changes relative to NBM of 10–40% and greater than 40% respectively.
(TIF)

Figure S5 Percentage of DMCs overlapping with repeats. Bar plots showing percentage of hyper- (magenta) and hypo-methylated (green) DMCs on repeat regions. Overall, 24–26% of hypo-methylated DMCs and ∼7% of hyper-methylated DMCs overlap with repeats. 10.7% of hypo-methylated DMCs of MLLr overlap with Alu repeats and 8.6% of hypo-methylated DMCs of IDH-mut overlap with Alu repeats.
(TIF)

Figure S6 DNA methylation and gene expression relationships display subtype-specific differences. CpG islands and shores across the genome were categorized into those located upstream from a transcription start site (TSS), overlapping a TSS or located downstream from a TSS. Boxplots are plotted that illustrate the maximum DNA methylation levels at these CpG islands and CpG shores for the high expressed genes (top 15th percentile expressed genes) and the low expressed genes (the bottom 15th percentile expressed genes). Each row is for a different sample: Normal bone marrow (top); IDH-mut AML (middle) and MLLr AML (bottom). The boxplots are color-coded depending on the expression status of associated genes. Significantly different distributions are marked with a star.
(TIF)

Table S1 Summary of RRBS and ERRBS experiments. All sequencing was performed using either the Illumina Genome analyzer II or HiSeq2000 (50 base pair, single reads). We routinely acquired >40 million reads per sample, with alignment rates ranging from 55–70%. Shown are the number of CpGs covered, bisulfite conversion efficiency and mean CpG coverage rates for each sample.
(DOCX)

Table S2 Methylation sequencing by MassARRAY EpiTYPER. MassARRAY was performed on bisulfite-converted DNA from HCT116 using the following primers targeting the listed amplicons.
(XLSX)

Table S3 Pathway analysis of DMCs in AML subtypes. Pathway enrichment analysis was performed using GREAT. Enriched terms in PANTHER Pathways are shown with their hyper-geometric test and binomial test q-values. (A) Pathway analysis for uniquely hyper-methylated DMCs in IDH-mut AML samples. (B) Pathway analysis for uniquely hypo-methylated DMCs in MLL-r AML samples. (See separate excel spreadsheet for full list of genes in each pathway).
(XLSX)

Table S4 Pathway analysis of concordantly hypermethylated DMCs in AML subtypes. Pathway enrichment analysis was performed using GREAT. Enriched terms in PANTHER Pathways are shown with their hyper-geometric test and binomial test q-values. Results from pathway analysis for concordantly hypermethylated DMCs in IDH-mut and MLL-r AML samples are listed.
(DOCX)

Table S5 Genes with recurrent aberrant DNA methylation by HELP that were validated by ERRBS. Listed are the fifteen (out of a total of eighteen) genes covered by both assays that were hypermethylated in the current study.
(DOCX)
