# Supplementary figures and images for: Base-Pair Resolution DNA Methylation Sequencing Reveals Profoundly Divergent Epigenetic Landscapes in Acute Myeloid Leukemia
Source: PLoS Genet. 2012 Jun 21;8(6):e1002781. doi: 10.1371/journal.pgen.1002781 (PMC3380828; doi:10.1371/journal.pgen.1002781)

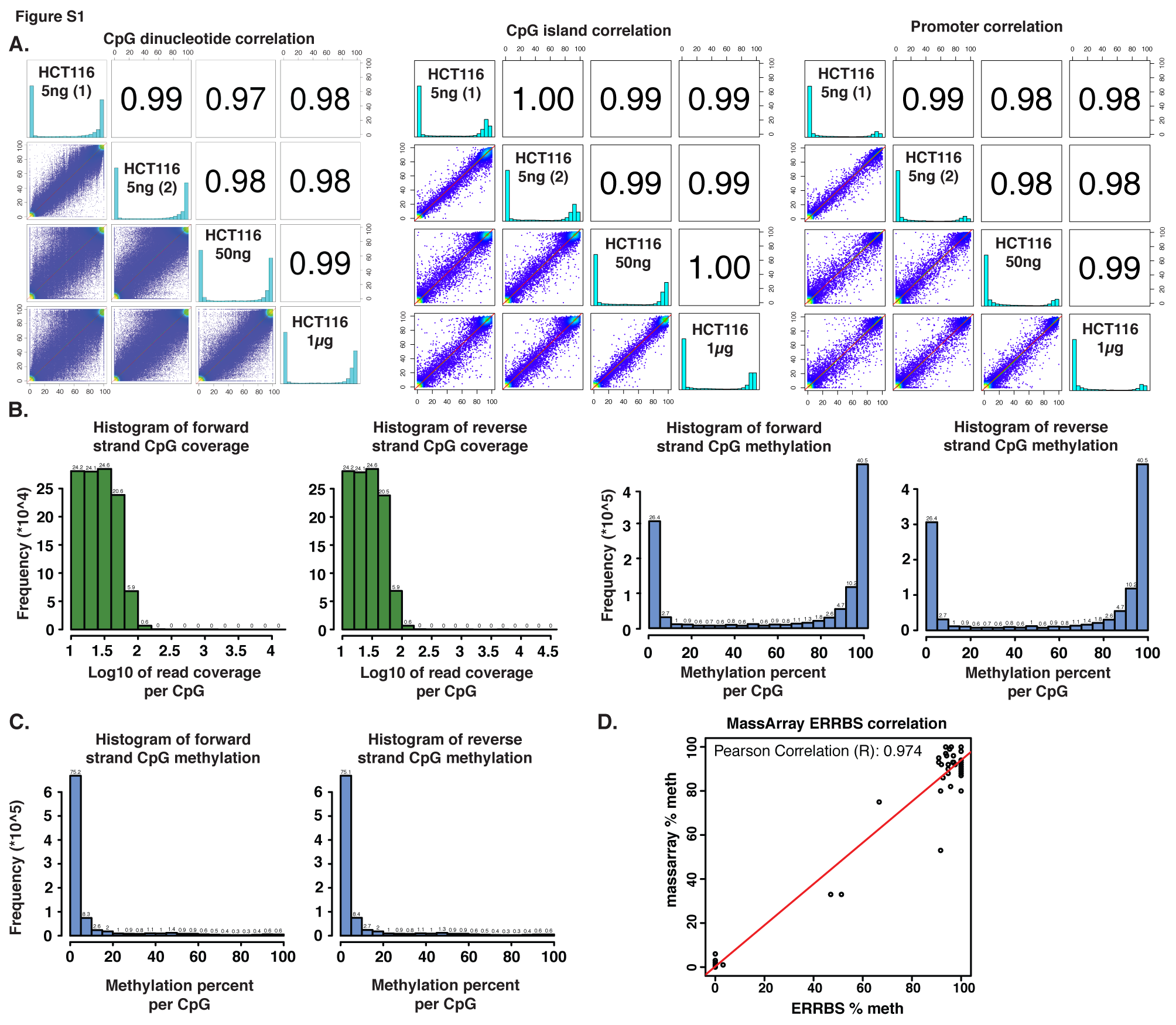

Supplement: Figure S1 — ERRBS is highly reproducible and sensitive. (A) Correlation between CpG dinucleotides, CpG islands and promoter methylation levels using pearson correlation between technical replicas of ERRBS using 5, 50 or 1000 ng genomic DNA from the HCT116 cell line. (B) Distribution histograms of CpG coverage and CpG methylation levels along forward and reverse strands in HCT116 ERRBS results. (C) Distribution histogram of CpG methylation levels along forward and reverse strands in DKO ERRBS results. Similar results were obtained from reverse strand (data not shown) and CpG coverage distributions over both strands were similar to coverage seen with HCT116 sequencing (data not shown). (D) Technical validation of ERRBS performance in HCT116 at select CpGs by MassARRAY. Dot plot shows correlation between DNA methylation as measured by ERRBS (x-axis) and percent methylation as measured by MassARRAY EpiTyper (y-axis). (Correlation coefficient: 0.97). (TIF) [file pgen.1002781.s001.tif]

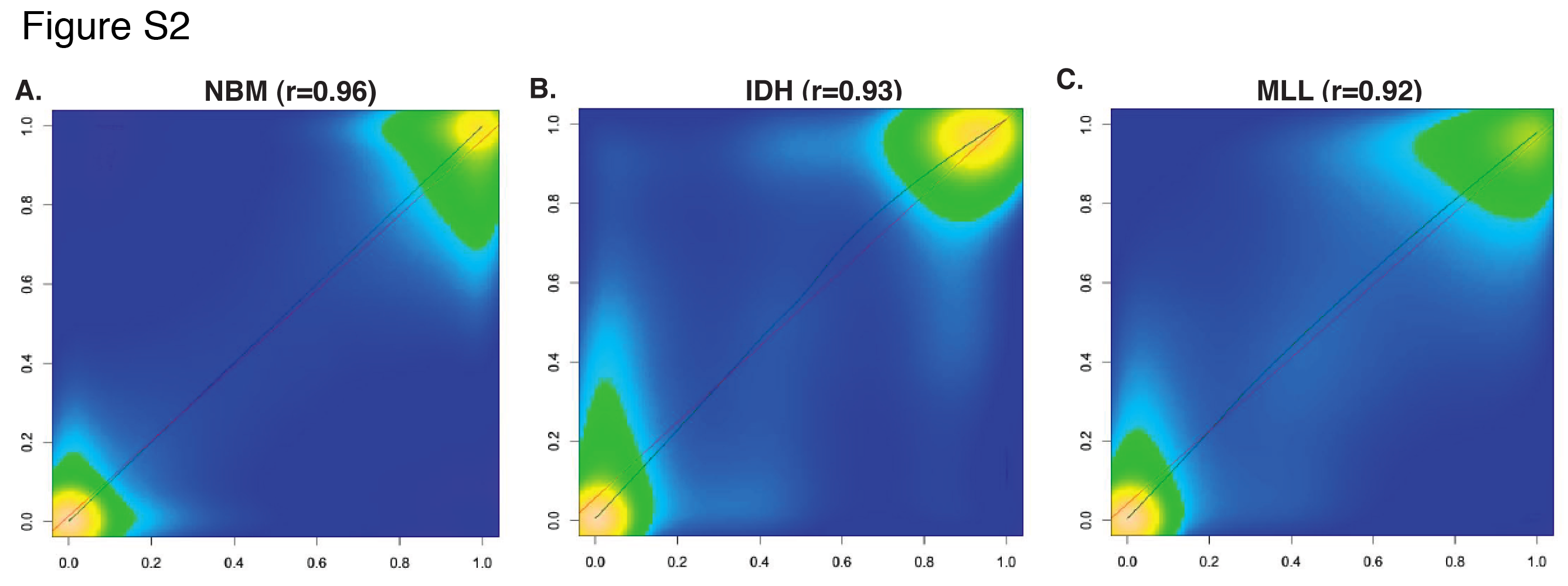

Supplement: Figure S2 — Biological replica reproducibility. (A) Correlation plot of CpG dinucleotide methylation levels between two biological replica of ERRBS data using normal bone marrow controls (NBM_#1 and NBM_#2_Rep#2). (B) Correlation plot of CpG dinucleotide methylation levels between two biological replica of ERRBS data using IDH mutant AML samples (IDH-mut_#1 and IDH-mut_#2). (C) Correlation plot of CpG dinucleotide methylation levels between two biological replicas of ERRBS data using MLL translocated AML samples (MLLr_#1_Rep#2 and MLLr_#2). (TIF) [file pgen.1002781.s002.tif]

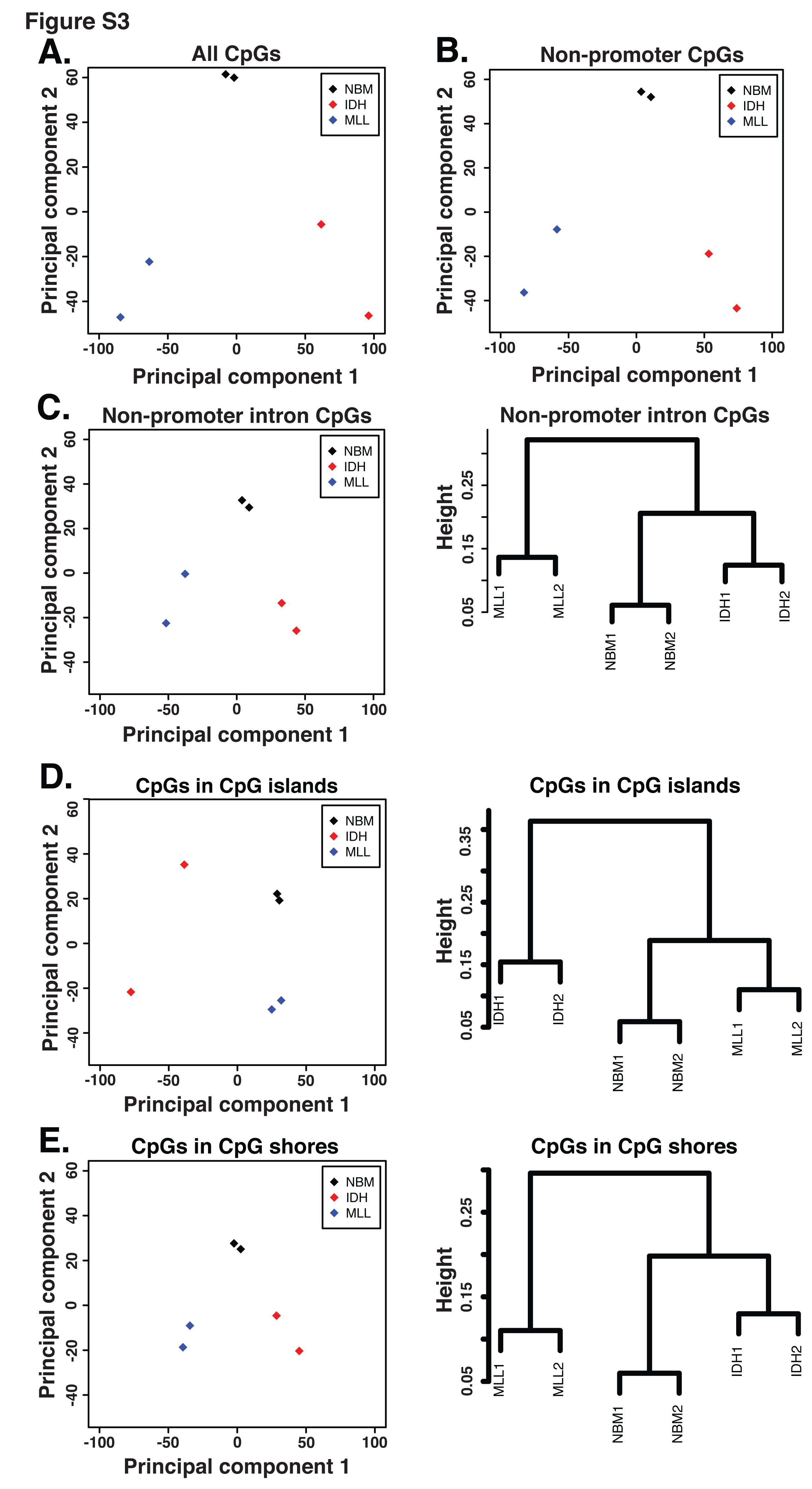

Supplement: Figure S3 — DNA methylation patterns naturally segregate AML and NBM samples. Unsupervised analysis using either principal component analysis or hierarchical clustering (1-Pearson correlation distance + Ward's agglomerative algorithm) of DNA methylation by ERRBS at (A) all CpGs, (B) non-promoter CpGs, (C) non-promoter intron CpGs, (D) CpGs within CpG islands and (E) CpGs within CpG shores, segregates the samples into their three biological groups. (TIF) [file pgen.1002781.s003.tif]

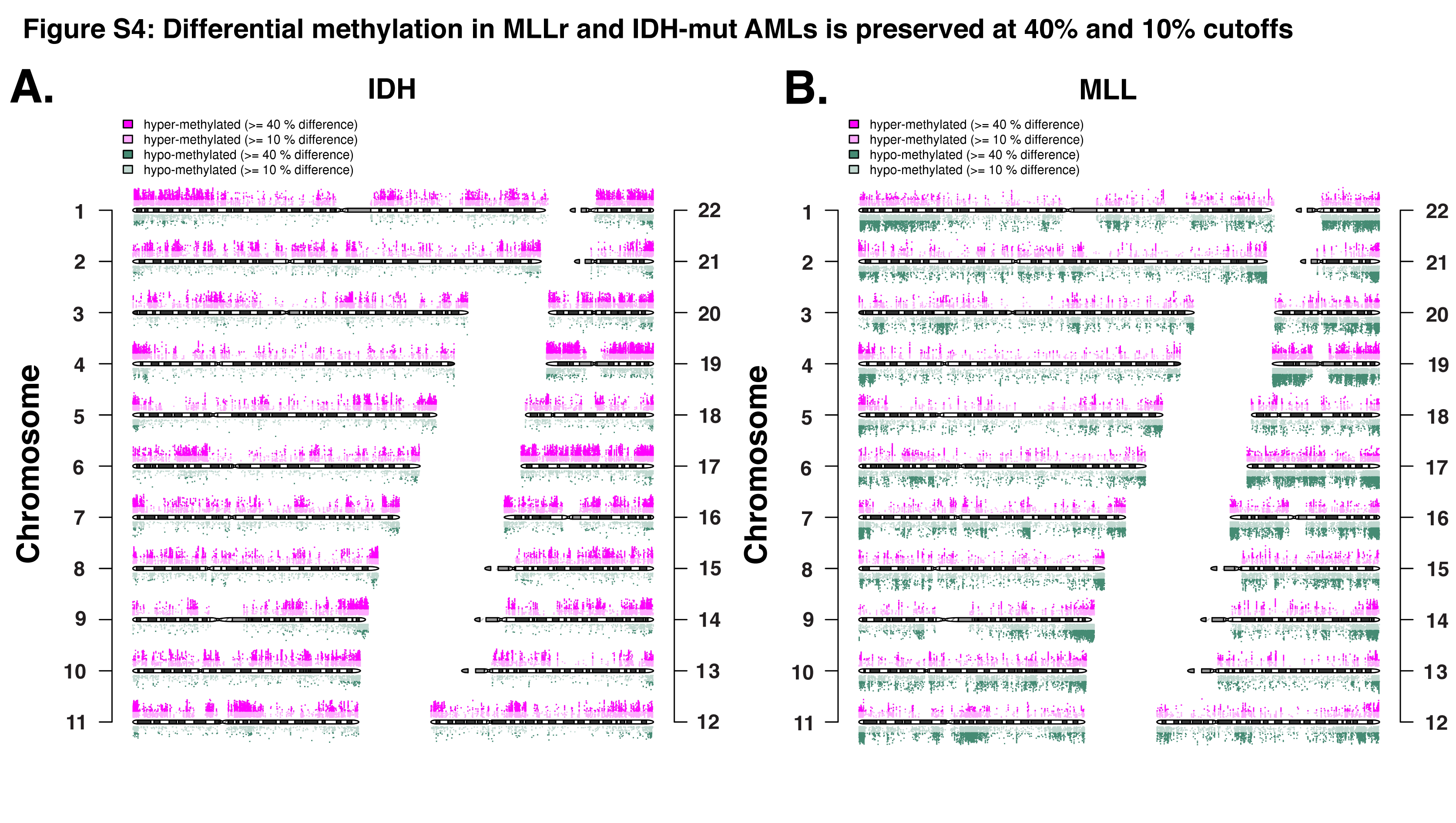

Supplement: Figure S4 — Differential methylation in MLLr and IDH-mut AMLs are preserved at 40% and 10% cutoffs. Chromosome ideogram representing differential methylation in IDH-mut AMLs vs. NBM (A) and MLLr AMLs vs. NBM (B), using changes greater than 10%. Light and dark magenta points represent hypermethylation changes relative to NBM of 10–40% and greater than 40% respectively. Light and dark green points represent hypomethylation changes relative to NBM of 10–40% and greater than 40% respectively. (TIF) [file pgen.1002781.s004.tif]

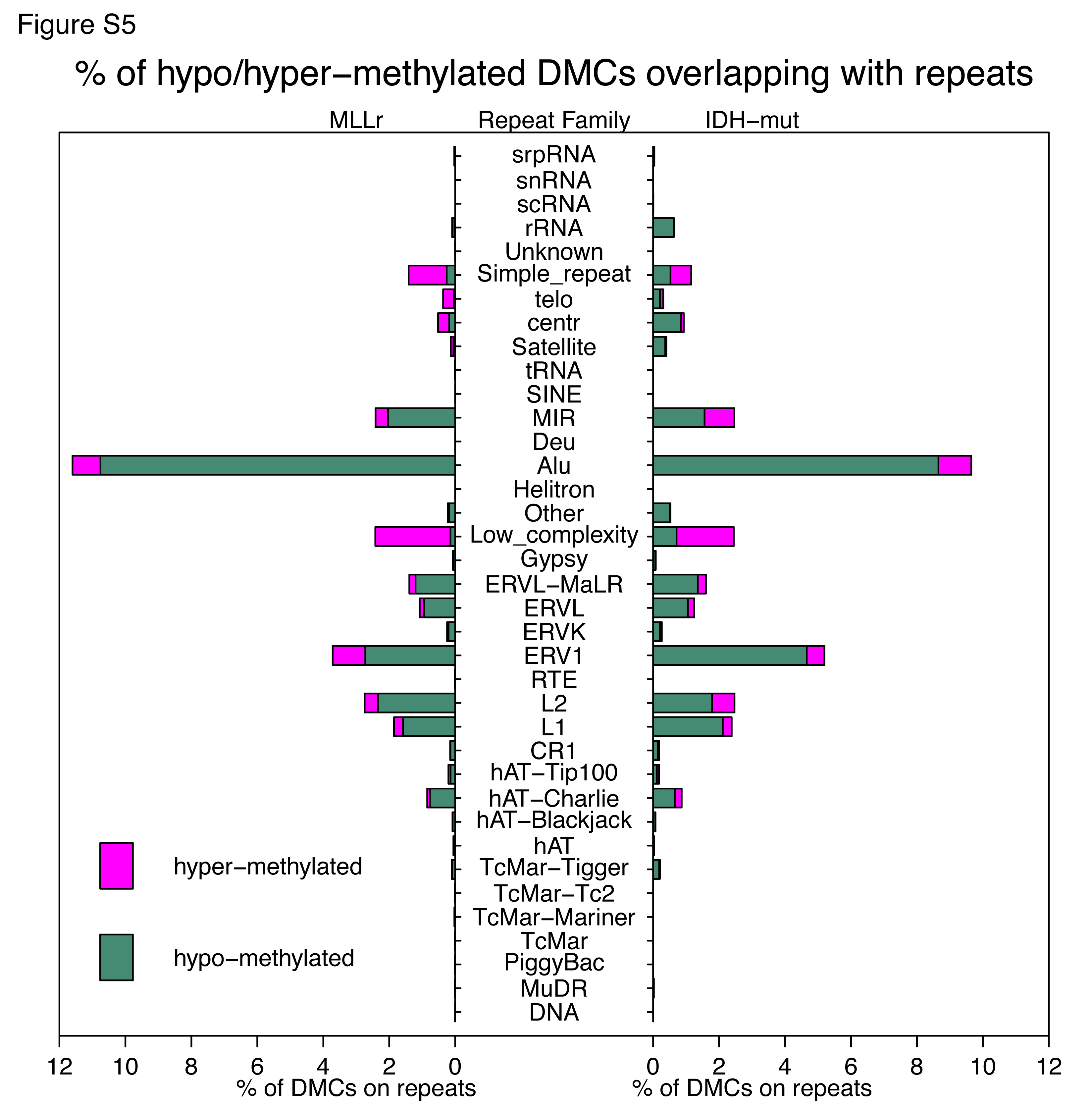

Supplement: Figure S5 — Percentage of DMCs overlapping with repeats. Bar plots showing percentage of hyper- (magenta) and hypo-methylated (green) DMCs on repeat regions. Overall, 24–26% of hypo-methylated DMCs and ∼7% of hyper-methylated DMCs overlap with repeats. 10.7% of hypo-methylated DMCs of MLLr overlap with Alu repeats and 8.6% of hypo-methylated DMCs of IDH-mut overlap with Alu repeats. (TIF) [file pgen.1002781.s005.tif]

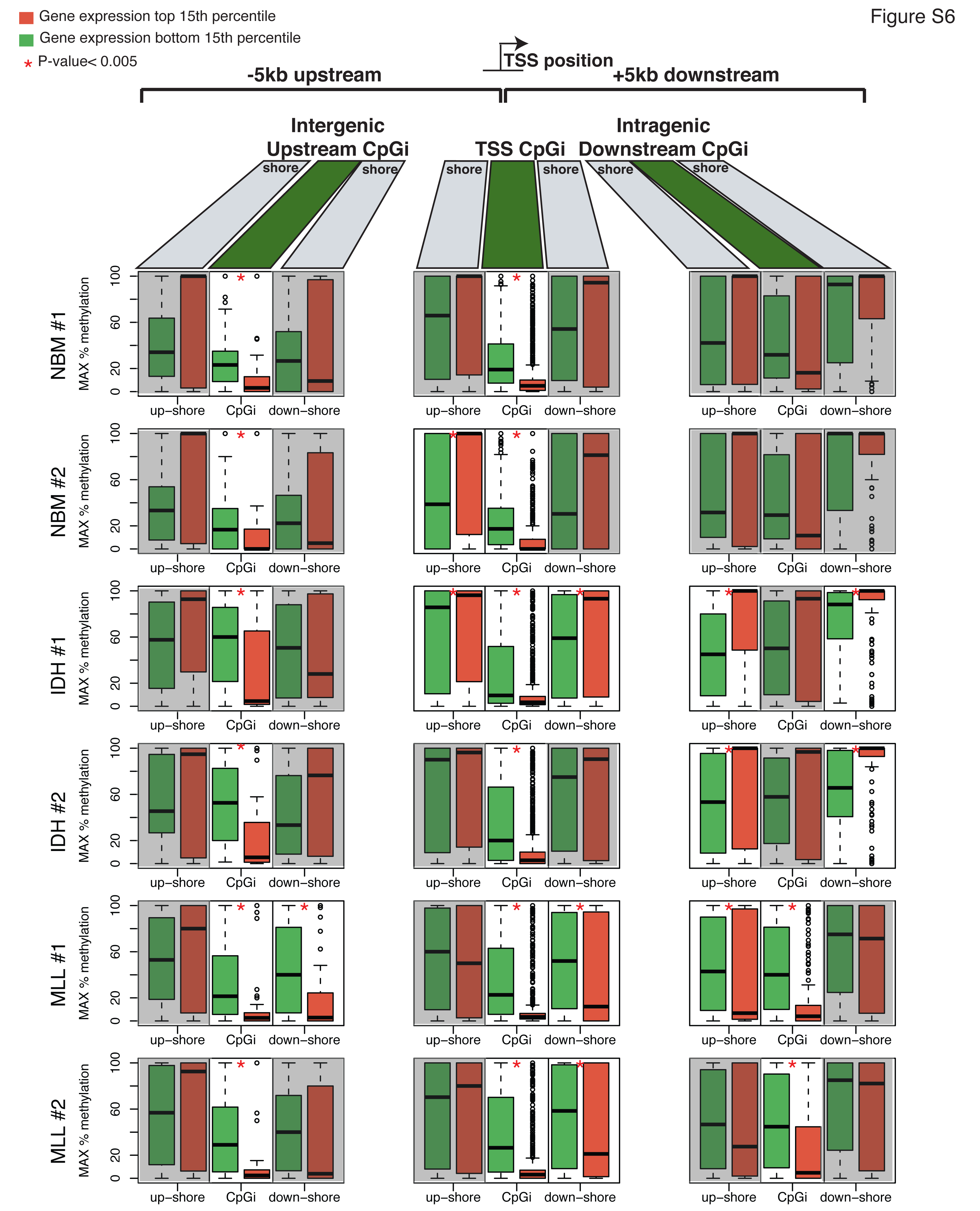

Supplement: Figure S6 — DNA methylation and gene expression relationships display subtype-specific differences. CpG islands and shores across the genome were categorized into those located upstream from a transcription start site (TSS), overlapping a TSS or located downstream from a TSS. Boxplots are plotted that illustrate the maximum DNA methylation levels at these CpG islands and CpG shores for the high expressed genes (top 15th percentile expressed genes) and the low expressed genes (the bottom 15th percentile expressed genes). Each row is for a different sample: Normal bone marrow (top); IDH-mut AML (middle) and MLLr AML (bottom). The boxplots are color-coded depending on the expression status of associated genes. Significantly different distributions are marked with a star. (TIF) [file pgen.1002781.s006.tif]
