# Supplementary material for: Base-Pair Resolution DNA Methylation Sequencing Reveals Profoundly Divergent Epigenetic Landscapes in Acute Myeloid Leukemia
Source: PLoS Genet. 2012 Jun 21;8(6):e1002781. doi: 10.1371/journal.pgen.1002781 (PMC3380828; doi:10.1371/journal.pgen.1002781)
Supplement: Table S1 — Summary of RRBS and ERRBS experiments. All sequencing was performed using either the Illumina Genome analyzer II or HiSeq2000 (50 base pair, single reads). We routinely acquired >40 million reads per sample, with alignment rates ranging from 55–70%. Shown are the number of CpGs covered, bisulfite conversion efficiency and mean CpG coverage rates for each sample. (DOCX) [file pgen.1002781.s007.docx]

Supplementary Table 1: Summary of RRBS and ERRBS experiments.

| Sample | Protocol | Total # of reads | Alignment rate | # of uniquely aligned reads | Bisulfite conversion rate (%) | # of CpGs (total) | Mean coverage per CpG |
| --- | --- | --- | --- | --- | --- | --- | --- |
| HCT116 5ng_Rep#1 | RRBS | 75228106 | 60% | 45145568 | 99.83% | 2279867 | 55 |
| HCT116 5ng_Rep#2 | RRBS | 37583520 | 57.90% | 21754705 | 99.81% | 1732224 | 35 |
| HCT116 50ng | RRBS | 125858552 | 64.70% | 12342255 | 99.65% | 2580644 | 57 |
| HCT116 50ng_ERRBS | ERRBS | 72983076 | 66.2% | 48287564 | 99.49% | 2320012 | 32 |
| HCT116 1µg | RRBS | 79593220 | 68.70% | 54687530 | 99.77% | 2077504 | 46 |
| DKO | RRBS | 67657535 | 67% | 45320521 | 99.90% | 1776890 | 64 |
| NBM_#1 ^ | ERRBS | 42207875 | 57.80% | 24396926 | 99.91% | 2405905 | 28 |
| NBM_#2 * | RRBS | 61485331 | 65.90% | 40547481 | 99.85% | 1088106 | 20 |
| NBM_#2_Rep#1 * | ERRBS | 136288178 | 68.40% | 93222324 | 99.85% | 2755435 | 22 |
| NBM_#2_Rep#2 *^ | ERRBS | 72951079 | 68.50% | 49999470 | 99.86% | 1447471 | 17 |
| MLLr_#1 * | ERRBS | 62818336 | 55.60% | 34909008 | 99.92% | 1995974 | 56 |
| MLLr_#1_Rep#1 * | RRBS | 69240235 | 69% | 47765870 | 99.80% | 1201862 | 42 |
| MLLr_#1_Rep#2 *^ | ERRBS | 132929572 | 70% | 93392491 | 99.82% | 2339523 | 41 |
| MLLr_#2 *^ | ERRBS | 76062433 | 69.40% | 52817564 | 99.82% | 1774753 | 31 |
| IDH-mut_#1 ^ | ERRBS | 78136444 | 65.20% | 50968373 | 99.87% | 2331754 | 50 |
| IDH-mut_#2 ^ | ERRBS | 33993656 | 64.90% | 22070235 | 99.84% | 1213687 | 37 |
| AML * | RRBS | 73151027 | 67.90% | 49674394 | 99.84% | 1291112 | 42 |
| AML_Rep#1 * | ERRBS | 121628994 | 67.30% | 81904566 | 99.82% | 2492806 | 36 |
| AML_Rep#2 * | ERRBS | 79232791 | 67.20% | 53253872 | 99.85% | 1744702 | 35 |

* samples used for RRBS versus ERRBS comparisons; ^ samples used for leukemia biology studies
