# Supplementary material for: Base-Pair Resolution DNA Methylation Sequencing Reveals Profoundly Divergent Epigenetic Landscapes in Acute Myeloid Leukemia
Source: PLoS Genet. 2012 Jun 21;8(6):e1002781. doi: 10.1371/journal.pgen.1002781 (PMC3380828; doi:10.1371/journal.pgen.1002781)
Supplement: Table S4 — Pathway analysis of concordantly hypermethylated DMCs in AML subtypes. Pathway enrichment analysis was performed using GREAT. Enriched terms in PANTHER Pathways are shown with their hyper-geometric test and binomial test q-values. Results from pathway analysis for concordantly hypermethylated DMCs in IDH-mut and MLL-r AML samples are listed. (DOCX) [file pgen.1002781.s010.docx]

**Supplementary table 4: Pathway analysis of concordantly hypermethylated DMCs in AML subtypes.**

| Term Name | Binomial FDR  Q-Val | Binomial Fold Enrichment | Binomial Observed Region Hits | Hypergeometric  FDR Q-Val | Hypergeometric  Fold Enrichment | Hypergeometric Observed Gene Hits | Hypergeometric  Total Genes |
| --- | --- | --- | --- | --- | --- | --- | --- |
| Cadherin signaling pathway | 2.56E-52 | 4.12 | 181 | 5.85E-06 | 2.27 | 44 | 124 |
| Wnt signaling pathway | 6.68E-40 | 2.72 | 243 | 0.0005 | 1.67 | 69 | 264 |
| Notch signaling pathway | 5.03E-13 | 3.93 | 46 | 0.04 | 2.42 | 14 | 37 |
