# Supplementary material for: Base-Pair Resolution DNA Methylation Sequencing Reveals Profoundly Divergent Epigenetic Landscapes in Acute Myeloid Leukemia
Source: PLoS Genet. 2012 Jun 21;8(6):e1002781. doi: 10.1371/journal.pgen.1002781 (PMC3380828; doi:10.1371/journal.pgen.1002781)
Supplement: Table S5 — Genes with recurrent aberrant DNA methylation by HELP that were validated by ERRBS. Listed are the fifteen (out of a total of eighteen) genes covered by both assays that were hypermethylated in the current study. (DOCX) [file pgen.1002781.s011.docx]

**Supplementary table 5: Genes with recurrent aberrant DNA methylation by HELP that were validated by ERRBS.**

| Accession | Gene symbol |
| --- | --- |
| NM_022103 | ZNF667 |
| NM_001124758 | SPNS2 |
| NM_001882 | CRHBP |
| NM_024705 | DHRS12 |
| NM_025163 | PIGZ |
| NM_178140 | PDZD2 |
| NM_183374 | CYP26C1 |
| NM_198569 | GPR126 |
| NM_001059 | TACR3 |
| NM_005953 | MT2A |
| NM_053024 | PFN2 |
| NM_024597 | MAP7D3 |
| NM_001137554 | MCTS1 |
| NM_000857 | GUCY1B3 |
| NM_012482 | ZNF281 |
